# A Centimeter-Scale Quadruped Piezoelectric Robot with High Integration and Strong Robustness

**DOI:** 10.34133/cbsystems.0340

**Published:** 2025-07-22

**Authors:** Yu Gao, Jing Li, Jie Deng, Shijing Zhang, Yingxiang Liu

**Affiliations:** State Key Laboratory of Robotics and System, Harbin Institute of Technology, Harbin 150001, China.

## Abstract

Centimeter-scale robots have unique advances such as small size, light weight, and flexible motions, which exhibit great application potential in many fields. Notably, high integration and robustness are 2 key factors determining the locomotion characteristics and practical applications. Here, we propose a novel centimeter-scale quadruped piezo robot. The robot’s locomotion is generated by multi-dimensional vibration trajectories at the feet, which are produced through a novel built-in actuation method. The robot achieves high locomotion speed (47.38 body length per second), high carrying capability (28.96 times self-weight), and high-resolution motion (minimum step size of 0.33 μm). Benefiting from the built-in integration method, the robot realizes the built-in integration of actuation, control, communication, and power supply, enabling untethered movement and strong robustness. It has a low startup voltage (10 *V*_0-p_) and an endurance time of 32 min. Furthermore, after enduring 3 consecutive drops, 2 kicks, and being stepped on by an adult (over 3,500 times its own weight), the system remains functional and continues to move afterward. The robot utilizes modular expansion to achieve image sensing applications, including multi-object image capture and object detection. This work provides inspiration for the balance between high-integration design and robustness in centimeter-scale robots.

## Introduction

Centimeter-scale robots have become a prominent research topic due to their unique advantages, such as small size, light weight, and high mobility. These features enable them to carry sensors or serve as mobile platforms to access narrow and challenging environments. Researchers have explored their applications in various fields, including micromanipulation [1,2], biotechnology [3,4], and environmental exploration [5,6]. This means that biomedical researchers or search-and-rescue teams have the opportunity to utilize centimeter-scale robots to carry out their tasks. Recent advancements in this domain have demonstrated the potential of centimeter-scale robots in high-performance locomotion. For example, the BHMbot [7] developed by Liu et al. achieves a maximum speed of 17.5 body lengths per second (BL/s) and flexible motion control through coil and flexible hinge transmission. Wang et al. [8] designed a miniature amphibious robot driven by eccentric motors, which can reach a speed of 815 mm/s (about 11 BL/s). The DASH [9] designed by Birkmeyer et al. achieves a movement speed of 15 BL/s using a single drive motor, with rigid linkages and polymer hinges. Although they have achieved certain advancements in miniaturization and motion performance, the presence of electromagnetic motors and transmission mechanisms prevents further miniaturization. There are still issues such as electromagnetic interference and wear of the transmission components.

In recent years, the development of smart materials brings new drive and transmission approaches in the fields of small robots, providing promising solutions for the miniaturization. Some functional materials such as dielectric elastomer materials, shape memory alloy materials, magnetostrictive materials, and piezoelectric materials have been applied in the field of small robots. The small robots driven by dielectric elastomers [10–12] are typically designed with simple structures, but require a high driving electric field (typically >1 × 10^5^ V/mm), which limits the widespread application. Small robots driven by shape memory alloys [13,14] exhibit a large recovery strain, but they typically have a long response time due to the slow temperature exchanging process. Magneto-elastic small robots [15,16] possess strong continuous deformation capabilities, while they require bulky setups to generate an external magnetic field, which increases the complexity of the whole robot system and restricts the range of movement. Moreover, some other small robots driven by light [17] and heat [18] have been developed, but they exhibit slow response speeds. Although these actuation schemes using smart materials offer approaches for robot miniaturization, they suffer some challenges in terms of excitation conditions, response characteristics, and untethered self-power locomotion.

Piezoelectric actuation [19–24], as an approach utilizing smart piezoelectric material to achieve motions, holds many advantages including fast response, simple structure and no transmission mechanisms. This actuation approach has already been used to develop small robots. Miniature piezoelectric robots can be classified into resonant and non-resonant types depending on whether they work in a resonant state. The resonant piezoelectric robots generally feature high speed and no motion noise. However, there are still important challenges in applying piezoelectric actuation to develop centimeter-scale robots, mainly including 2 aspects. (a) Relatively poor robustness. Piezoelectric ceramics are brittle materials, and piezoelectric robots typically feature a metal substrate structure with external piezoelectric ceramics; (b) Low integration of the robot. The actuation, control, communication, and power supply units are generally separate. The complex drive and control unit is typically located directly above the robot, presenting challenges in module packaging and raising the center of gravity.

Therefore, centimeter-scale robots with high integration and strong robustness demonstrate potential for practical applications. Here, we propose a novel centimeter-scale quadruped piezoelectric robot with high integration and strong robustness, which promises to bring new perspectives for the construction and application of centimeter-scale robots.

This work mainly achieves 4 key advancements: (a) A new built-in actuation method is proposed, and a centimeter-scale tethered robot prototype (14.47 g, 70 mm × 13 mm × 15.8 mm) is designed. It achieves fast locomotion with a speed up to 47.38 BL/s, a high carrying capability of 28.96 times self-weight, and a high resolution of 0.33 μm. The robot can perform cross-scale movement from sub-micron (micro) to meter (macro) level; (b) The untethered centimeter-scale robot realizes the integration of actuation, control, communication, and power supply through the built-in integration method, ensuring all functional units are protected within the metal substrate of the piezo unit. It has a low startup voltage (10 V_0-p_) and an endurance time of 32 min; (c) After being stepped on by an adult human’s full body weight (66.45 kg, over 3,500 times heavier than the robot), as well as enduring 3 consecutive drops and 2 kicks, the robot still operates normally and continues to move afterward, demonstrating exceptional robustness; (d) The robot with a sensor module achieves real-time image grayscale conversion, multi-object image capture, color block tracking, and object detection, exhibiting great potential for image sensing applications and modular expansion. The proposed new built-in actuation method and the built-in integration method provide a design reference for high integration and strong robustness in centimeter-scale robots.

## Materials and Methods

### Robot materials and fabrication

The built-in actuation robot is made of aluminum alloy and 8 pieces of PZT-4 piezoelectric ceramic plates. The connectors and the shells are fabricated by 3-dimensional (3D) printing technology (stereo lithography appearance, photosensitive resin). For detailed material parameters, please refer to the Supplementary Materials (see Note S2). The fabrication process of the robot is shown in Fig. S9A. We first fix a piece of piezoelectric ceramic on the desktop and apply epoxy resin glue to one side, ensuring that the glue is applied evenly and in a small amount [see Fig. S9A(a) and (b)]. The piezoelectric ceramic is pushed into the robot body on one side [Fig. S9A(c)]. Ensure that the piezoelectric ceramic is as flush as possible with the robot end face (the distance error < 1.5 mm). The same is true on the other end. Then, we use a pressing plate (computerized numerical control, aluminum, with threaded holes in the center), clamping block (3D printed), and preload screw for fixation and preloading. By rotating the preload screw, an appropriate preload force can be applied to the piezoelectric ceramic, ensuring a tight bond between the ceramic piece and the metal surface [see Fig. S9A(d)]. Similarly, 8 pieces of piezoelectric ceramics on all 4 sides can be installed and preloaded [see Fig. S9A(e)]. After standing at room temperature (25 °C) for 24 h, the epoxy resin adhesive will fully cure. After removing the preload mechanism, use the solder wire and soldering iron to fix the wire onto the surface of the silver layer. It should be noted that the solder joints are welded at both ends of the robot, with the wire directed inward [see Fig. S9A(f), the image is shown with the wire directed outward for easier observation]. This arrangement facilitates the integration and wiring of the wireless motion system. Fig. S9B describes the materials used in the robot’s construction, including the metal substrate and piezoelectric ceramics.

### Experimental design and data analysis

We build a robot experimental test system as shown in Fig. S10. The test subjects include the built-in actuation robot, the built-in integration robot, and the dual-module sensing robot (DMSR). The built-in actuation robot is stimulated by the applied voltage through wires. Unless otherwise specified, all tests are conducted on a transparent glass substrate (600 mm × 600 mm × 5 mm). In addition, modular expansion and image sensing application experiments are also tested on this platform. We use a 30-fps with 1,080P resolution high-definition USB camera (Hikvision, China) connected to a tripod for overhead shooting to record the trajectory of the robot. The excitation signal required for the tethered test is provided by the switching power supply (QD-8D, China). The untethered locomotion test of the robot is a start–stop command sent through a mobile phone. All speed characteristic test videos are analyzed by Adobe Premiere Pro and the average speed is calculated by recording the relative positions of the robot at keyframes. All calculations are performed by the self-developed speed calculation software (Python and PyQT5).

In the speed characteristics testing experiment, the error bars on the data points represent the measurement deviations from 3 repeated experiments. Statistical analysis of the data is conducted using OriginPro 2024b.

## Results

### Structural design and locomotion analysis

**Fig. 1.**
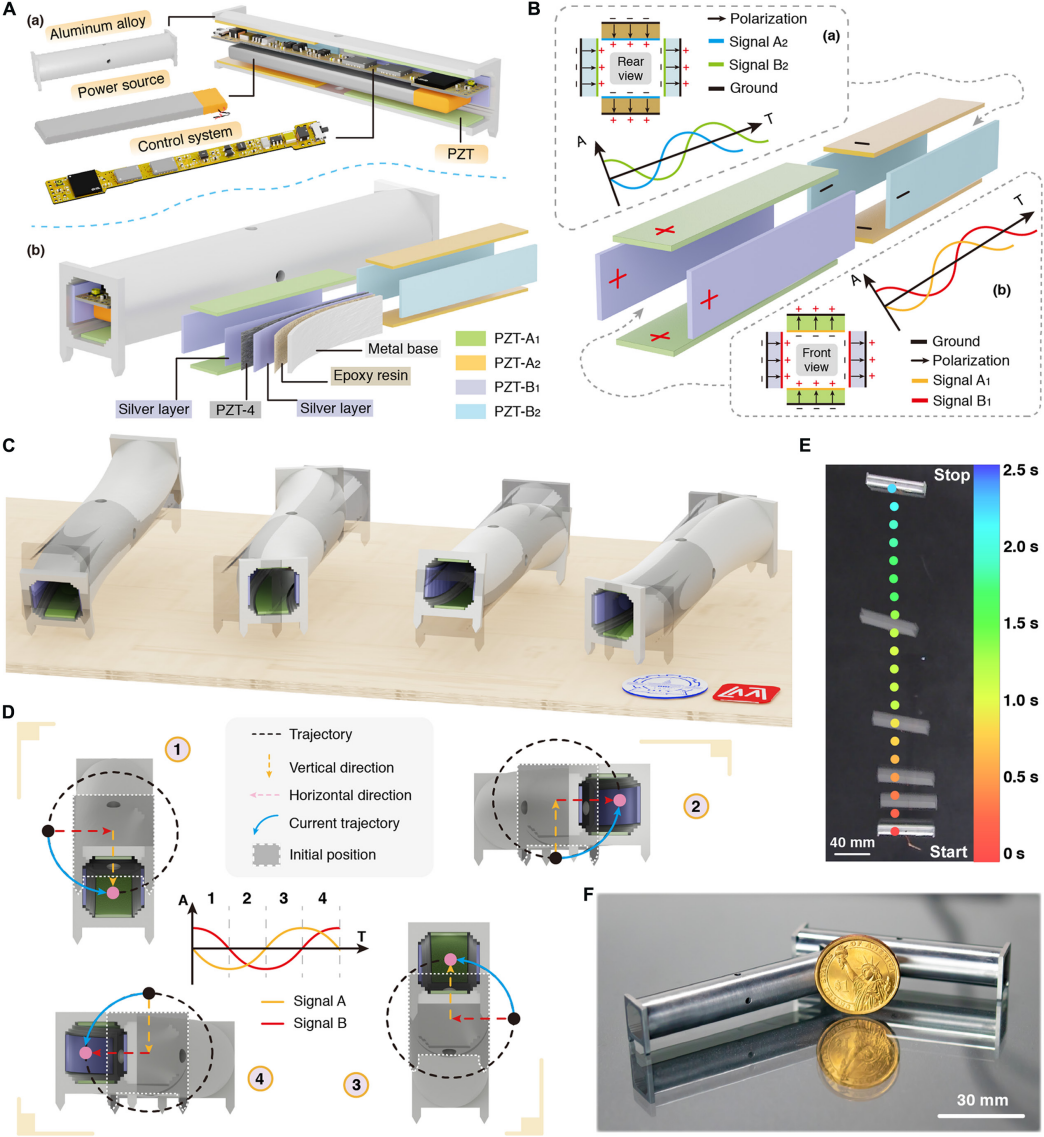
Design and working principle of the robot. (A) The structure of the robot. (a) The robot includes the piezoelectric ceramics, metal body, power source, and control system. (b) The arrangement and structure of piezoelectric ceramics. (B) Signal distribution, spatial arrangement, and polarization directions of the piezoelectric ceramics (the positive and negative signs only indicate the polarization direction, independent of the electrode). (C) The 4 consecutive key states of the robot in one motion cycle. (D) Generation strategy of the multi-dimensional elliptical vibration trajectory at the foot end. (E) Series of optical images recording the linear motion process of the prototype robot. (F) A photo of the 2 robots’ metal bodies, alongside a 1-dollar coin.

The proposed miniature quadruped piezo robot consists of piezoelectric ceramics, a metal body (Al, 2A12), a power source, and a control system, as shown in Fig. 1A(a). The robot achieves untethered movement at the centimeter scale (18.8 g, 7 cm × 1.3 cm × 1.58 cm, untethered), and all components are located inside the metal body (see Movie S1). There are 8 pieces of piezoelectric ceramics, divided into 2 groups (horizontal group: PZT-A_1_ and PZT-A_2_ and vertical group: PZT-B_1_ and PZT-B_2_), as shown in Fig. 1A(b). Each PZT element is plated with silver on the both sides for applying exciting signals conveniently and is pasted to the inside body base using epoxy resin. The robot features 4 through-holes and 4 long strip-shaped drive feet, which facilitate modular expansion and support. The design principles of the driving feet mainly consider the following aspects. (a) Minimal impact on the robot body vibrations. The existence of the driving feet needs to minimize the influence on the main structure and resonant frequency; (b) Lightweight. The driving feet should avoid complex structures and excessive weight.

The polarization directions of the 2 opposite PZT plates are the same, while the polarization directions of adjacent PZT plates are opposite, as shown in Fig. 1B. This polarization scheme and the arrangement of the piezoelectric ceramics simplify the number of excitation signals (see Note S1). The robot only needs 2 types of sinusoidal signals to achieve linear and turning movements (signal A: PZT-A_1_ and PZT-A_2_; signal B: PZT-B_1_ and PZT-B_2_). Signal A_1_ and signal A_2_ only differ in amplitude to control the turning locomotion, while signal A and signal B have the same frequency but a 90° phase difference, which facilitates the synthesis of the elliptical trajectory. The entire metal body is grounded.

The robot achieves noiseless motion through ultrasonic resonant actuation at frequencies beyond the human auditory range (>20 kHz), making it particularly suitable for human–machine interaction applications. The built-in actuation method places piezoelectric ceramics inside the hollow metal body, generating alternating multi-dimensional elliptical vibration trajectories at both ends. It provides the periodic frictional forces among the driving feet and the working surface, as shown in Fig. 1C. The built-in integration method achieves centimeter-level integration of all functional units by miniaturizing the wireless motion system and embedding the actuation, control, communication, and power supply units inside the body. These 2 built-in methods can protect the actuation source and obtain a small and compact structure, which provides the foundation for combining the high-integration design and strong robustness at the centimeter level. Besides, the built-in structure provides a more compact and light structure for the robot and the hollow structure substantially helps reduce resonant frequency and increase resonant amplitude.

The elliptical trajectory at the foot end is synthesized for actuation by exciting the horizontal and vertical groups of piezoelectric ceramics to form periodic motion in the vertical and horizontal directions (see part I in Movie S2). Take one motion cycle as an example, as shown in Fig. 1D. Considering only linear motion, signal A_1_ and signal A_2_ are identical.

1. When *t* = 0 to *T*/4, the horizontal group ceramics changes from no deformation to maximum deformation, and the vertical group ceramics gradually changes from maximum deformation to no deformation. The combined displacement is 1/4 ellipse, and the voltage of PZT-B_1_ and PZT-B_2_ is 0.
2. When *t* = *T*/4 to *T*/2, the horizontal group ceramics changes from maximum deformation to no deformation, and the vertical group ceramics gradually changes initial state to maximum deformation. The robot is lifted upwards to present a horizontal bend, and the voltage of PZT-A_1_ and PZT-A_2_ is 0.
3. When *t* = *T*/2 to 3*T*/4, the horizontal group ceramics produce vertical displacement and gradually reach the maximum deformation, and the vertical group ceramics gradually produce horizontal displacement and return to the initial state. When *t* = 3*T*/4, the voltage of PZT-B_1_ and PZT-B_2_ is 0.
4. When *t* = 3*T*/4 to *T*, the horizontal group ceramics gradually changes from maximum deformation to no deformation, and the vertical group ceramics reaches the maximum deformation. The robot completes a complete counterclockwise elliptical motion trajectory, and the voltage of PZT-A_1_ and PZT-A_2_ is 0.

After the above periods, the elliptical trajectory can be obtained at the foot end. The robot operates in a second-order bending vibration mode, with the deformation at both ends being symmetric about the center. By changing the phase difference between the 2 signal groups (phase difference: +90° or −90°), the direction of the elliptical rotation is adjusted, thereby changing the locomotion direction of the robot. By altering the voltage amplitude between signals of the same group (either signal A_1_ and signal A_2_ or signal B_1_ and signal B_2_), the robot can perform turning locomotion. Part II in Movie S2 shows the principle animation of the linear and steering motion.

Benefiting from the built-in actuation method, the built-in actuation robot achieves fast locomotion with a speed of up to 47.38 BL/s. The sequence of motion frames of the robot on the glass plate is shown in Fig. 1E. The metal body of the robot is shown together with a 1-dollar coin for size comparison, as shown in Fig. 1F. The robot features a small size, light weight, and compact structure. The structural parameters and weight of each unit of the robot are shown in Fig. S2.

### Simulation analysis and performance characterization

**Fig. 2.**
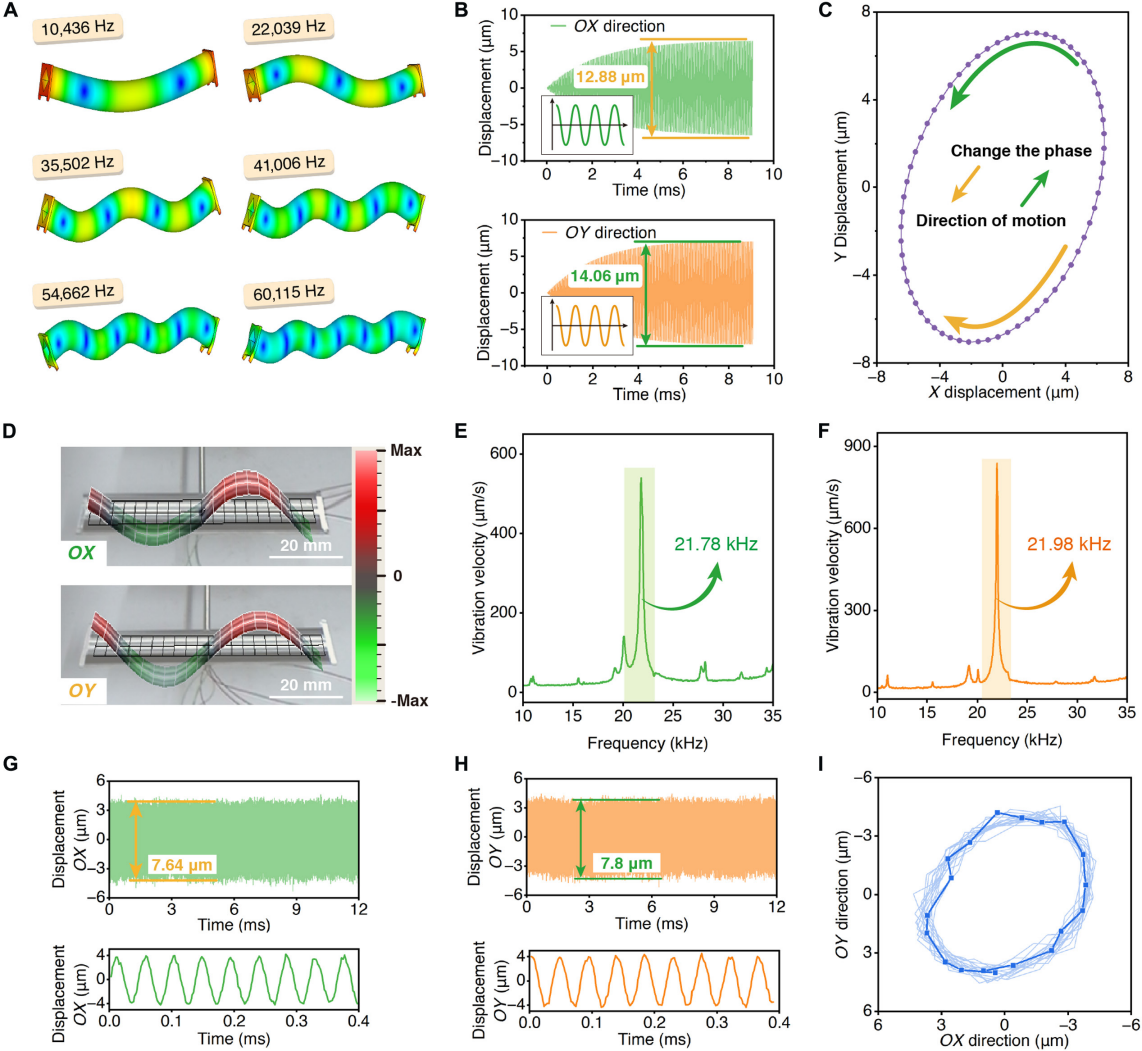
Robot simulation analysis and vibration characteristics. (A) The bending vibration shapes (modes 1 to 6) and corresponding frequencies of the robot. (B) Transient dynamics simulation results of the robot’s foot end by the finite-element method (*OX*: 12.88 μm, *OY*: 14.06 μm). (C) The foot end exhibits an elliptical motion trajectory in the *XOY* plane. (D) Second-order bending vibration modes in the *OX* and *OY* directions (red and green represent the outward and inward deformation of the tested position perpendicular to the measured surface, respectively). (E) and (F) represent the vibration characteristics test results in the *OX* and *OY* directions, respectively (*OX*: 21.78 kHz, *OY*: 21.98 kHz, 20 *V*_p-p_). (G) and (H) show the vibration displacements of the foot end in the *OX* and *OY* directions, respectively (*OX*: 7.64 μm, *OY*: 7.8 μm, 60 *V*_p-p_). (I) The vibration trajectory of the prototype foot end is approximately elliptical.

Simulation analysis plays an important role in verifying the feasibility of motion, and determination of parameters is conducted using the finite element method based on the following 3 principles. (a) The frequency of the horizontal (*OX*) and vertical (*OY*) vibration modes should be as close as possible, with a difference of no more than 3%; (b) Resonance frequency should be greater than 20 kHz to ensure that there is no noise during motion, but it should not be too high; (c) There must be an effective displacement at the foot end. Specifically, the displacement should be greater than 1 μm, which can ensure that the robot can overcome the frictional force between the foot end and the working surface. Furthermore, we analyze the high-efficiency excitation approach of the foot trajectories. The first 6 orders bending vibration modes of the robot are shown in Fig. 2A, along with their corresponding resonance frequencies (average frequency of the *OX* and *OY* directions). In vibration theory, lower-order modes are generally more efficient at acquiring energy and generating vibration compared to higher-order modes, and higher-order modes have more complex vibration shapes. In addition, the higher-order modes required more piezoelectric ceramics, as well as more complex manufacturing process. Therefore, we choose the second-order bending vibration mode as the working mode, and its resonance frequency is 22,039 Hz (*OX*: 22,025 Hz, *OY*: 22,053 Hz, difference: 0.13%). The transient simulation results of the robot under 160 *V*_p-p_ are shown in Fig. 2B. The amplitudes of driving foot vibration in *OX* direction and *OY* direction are 12.88 and 14.06 μm, respectively. The foot end exhibits an elliptical motion trajectory in the *XOY* plane, as shown in Fig. 2C. In fact, the robot also elongates in the axial direction (*OZ*), and the actual trajectory is an elliptical path in 3D space. However, since the displacement directions at both ends are opposite, only the *XOY* plane needs to be considered.

The prototype is processed after determining the parameters. First, the vibration characteristics are tested using the vibrometer test system (see Fig. S3), as shown in Fig. 2D. The test method can be seen in Note S3. Then, the *OX* and *OY* direction bending vibration modes are tested respectively, and the results are shown in Fig. 2E and F. The robot exhibits a second-order bending vibration mode and high vibration velocity (see Movie S3), with frequencies of 21.78 and 21.98 kHz, respectively. The difference between the 2 directions is only 0.9%. Compared with the simulation, the average frequency difference of the second-order bending vibration mode is only 0.73%, which validates the effectiveness of the design and fabrication.

To verify the working principle of the robot, a vibration displacement testing system is used to measure the motion trajectory of the driving foot (see Fig. S4). The displacement data in the *OX* direction are obtained when the vertical group ceramics (4 pieces, PZT-B_1_ and PZT-B_2_) is excited, and similarly, the data in the *OY* direction are also obtained. At a voltage of 60 *V*_p-p_ and a frequency of 21.98 kHz, the displacement in the *OX* and *OY* directions is 7.64 and 7.8 μm, respectively, as shown in Fig. 2G and H. An elliptical trajectory in the *XOY* plane has been successfully synthesized by selecting the points with less noise and relatively stable periods, as shown in Fig. 2I.

### Experiments of the robot

#### The built-in actuation robot

**Fig. 3.**
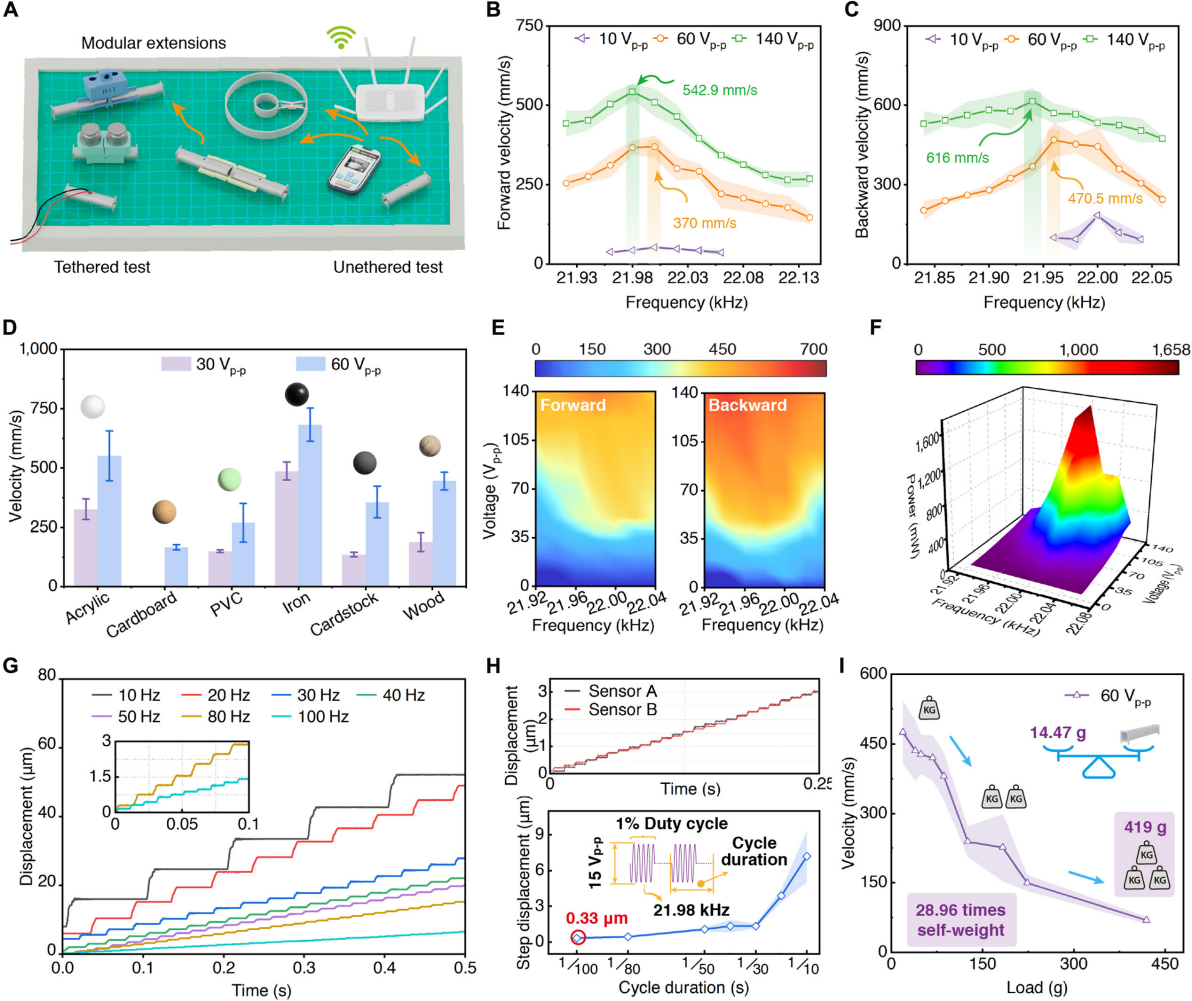
The locomotion characteristics of the built-in actuation robot. (A) Locomotion characteristic testing platform (including tethered test, untethered test and modular expansion) (details are found in Materials and Methods). (B) and (C) represent the relationship between velocity and frequency for forward and backward locomotion of the built-in actuation robot, respectively (forward: 542.9 mm/s, 21.98 kHz; backward: 616 mm/s, 21.94 kHz, 47.38 BL/s). (Note 1: In the experiments, each data was tested 3 times, and the mean and standard deviation were calculated to plot the error bars. Note 2: The external power supply uses an ultrasonic power source, which outputs a square wave. *V*_p-p_ = 2 *V*_0-p_ = 2 *U*_m_, *V*_rms_ = *U*_m_.) (D) The speed characteristic tests of the built-in actuation robot on 6 different substrates. (E) The relationship between velocity and voltage of forward and backward locomotion. (F) Power consumptions (10 to 140 *V*_p-p_) (maximum value: 1.658 W, 140 *V*_p-p_). (G) The relationship between pulse frequency and step displacement of robots in high-resolution mode (10 to 100 Hz, 1% duty cycle, 15 *V*_p-p_, 21.98 kHz). (H) The robot achieves an optimal resolution of 0.33 μm during a 1/100 cycle duration. (Note: The final result is obtained by taking the average of 2 displacement sensors placed in parallel, and the test is repeated 3 times.) (I) Locomotion characteristics under different loads (maximum value: 419 g, 28.96 times self-weight).

To verify the feasibility of the built-in actuation method and the built-in integration method, we develop the built-in actuation robot (tethered, 7 cm × 1.3 cm × 1.58 cm, 14.47 g) and place the wireless motion board and battery inside the metal substrate of the robot to fabricate a built-in integration robot (untethered, 18.8 g), which achieves untethered motion at the same size. The locomotion characteristics testing platform is established, as shown in Fig. 3A. The characteristics of forward and backward movements are shown in Fig. 3B and C, respectively. As the excitation frequency increases, there exists a peak speed, consistent with its resonant working principle. The built-in actuation robot demonstrates excellent speed performance (see part I in Movie S4), reaching maximum speeds of 542.9 mm/s forward and 616 mm/s backward (about 47.38 BL/s), respectively. The speed characteristics of forward and backward locomotion are basically consistent. The voltage of the purple curve represents the minimum startup voltage for the robot, and 10 *V*_p-p_ is sufficient for effective locomotion, which provides the foundation for the implementation of the built-in integration method. By adjusting the voltage amplitude between the ceramic groups at both ends (Group 1: PZT-A_1_ and PZT-B_1_ and Group 2: PZT-A_2_ and PZT-B_2_; *V*_Group1_ > *V*_Group2_, clockwise; *V*_group1_ < *V*_group2_, counterclockwise). There is a difference in the vibration amplitude of the driving feet at both ends, enabling turning motions (see part II in Movie S4). The vibrations of both ends are non-decoupled, which results in the robot being unable to perform rotational movement.

The robot achieves effective movement on 6 different substrates (see part III in Movie S4). Experimental results show that the built-in actuation robot possesses good adaptability to various contact working surfaces, with the fastest speed on the iron plate and the slowest on a cardboard and a polyvinyl chloride sheet, as shown in Fig. 3D. This facilitates the deployment of the robot on workbenches with varying surface materials. At the same frequency, the higher the excitation voltage, the larger the vibration amplitude of the piezoelectric ceramics. Similarly, the robot speed also increases with the rise in voltage, as shown in Fig. 3E. After testing the basic motion characteristics, power consumptions are tested by using a digital power meter (WT210, Yokogawa, Japan) at different voltages (10 to 140 *V*_p-p_), as shown in Fig. 3F. The maximum power of the built-in actuation robot’s forward motion is 1.658 W (8 pieces of PZT, 140 *V*_p-p_).

Under high-frequency continuous excitation conditions, the robot achieves meter-level high-speed motion (macro). Through high-frequency pulse excitation, the robot can perform continuous small-step motion (micro). Using the motion resolution test system (see Note S5), the step results are tested at pulse frequencies ranging from 10 to 100 Hz, as shown in Fig. 3G. The high-frequency pulse refers to a high-frequency sine wave signal with a certain duty cycle. In the test, the duty cycle is 1%, the excitation voltage is 15 *V*_p-p_, and the frequency of the high-frequency sine wave is 21.98 kHz. The results are averaged from data of 2 capacitive displacement sensors to eliminate errors. The example data from both sensors, as well as the relationship between the pulse signal’s cycle duration and step size, are shown in Fig. 3H. The minimum step size of the built-in actuation robot reaches 0.33 μm (cycle duration: 1/100 s), achieving sub-micron precision. The robot simultaneously supports high-resolution mode and high-speed motion mode, which means it can perform cross-scale movement from sub-micron (micro) to meter (macro) level.

We use 3D printing to create a loading platform, which is installed in the robot’s reserved through-hole, and conduct a load-carrying test (see part IV in Movie S4), as shown in Fig. 3I. As the load increases, the speed decreases (with a constant voltage 60 *V*_p-p_). The maximum load capacity is 419 g, which is equivalent to 28.96 times its own weight. Notably, the speed remains above 400 mm/s even with a load of 67.7 g, which means the robot can integrate wireless power and sensor modules to expand its range of motion and application scenarios.

#### The built-in integration robot

**Fig. 4.**
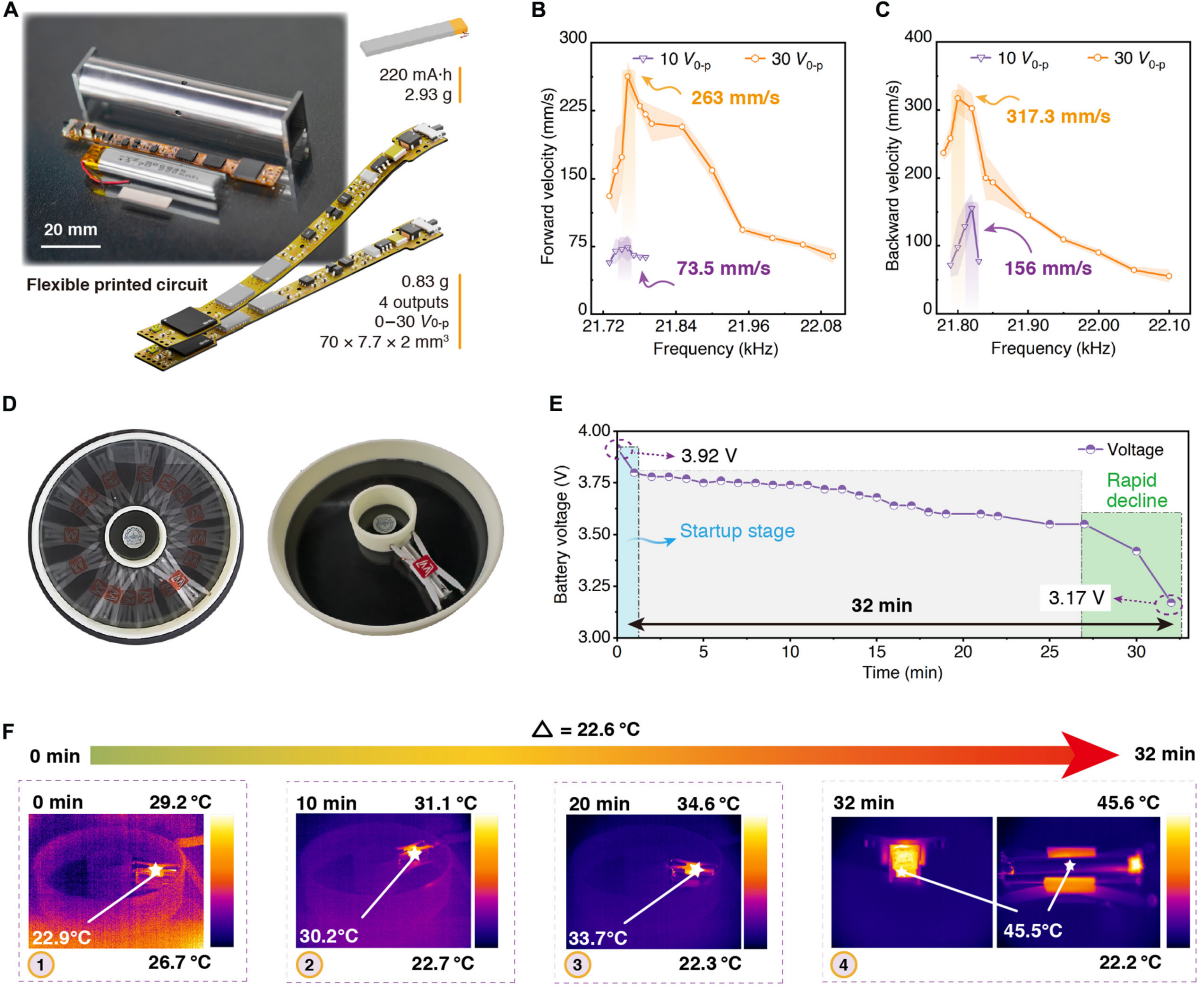
The locomotion characteristics of the built-in integration robot. (A) Photos and related parameters of the built-in integration robot prototype components. (B) and (C) represent the relationship between velocity and frequency for forward and backward locomotion of the built-in integration robot, respectively (forward: 263 mm/s, 21.76 kHz; backward: 317.3 mm/s, 21.80 kHz, 24.4 BL/s). (Note: Since the boost circuit ignores negative voltage output, the circuit does not produce a negative half-cycle. *V*_p-p_ = *V*_0-p_ = *U*_m_, *V*_rms_ ≈ 0.707 *U*_m_.) (D) Photos of the robot conducting endurance tests in a circular track. (E) The continuous movement time of the robot can reach 32 min (initial voltage: 3.92 V). (F) The robot moved continuously for 32 min, with a temperature rise of 22.6 °C and a maximum temperature of 45.5 °C.

The built-in integration robot achieves untethered locomotion with the proposed built-in integration method (see part I in Movie S5). The circuit board and battery are placed inside the metal body. This method achieves miniaturization of the wireless motion system and the built-in integration of actuation, control, communication, and power supply units at the centimeter level. The components of the built-in integration robot are shown in Fig. 4A. The control board uses a flexible printed circuit (70 mm × 7.7 mm × 2 mm), with a total weight of only 0.83 g and 4 independent output channels. Its detailed working principle and circuit diagram can be found in Note S6 and Fig. S6. The control chip is ESP32-PICO-D4 (Espressif, China), which provides communication (2.4 GHz Wi-Fi) and control signals. The circuit board can achieve 4 independent square wave signal outputs, with a maximum output voltage of 30 *V*_0-p_. The output square wave has adjustable voltage and frequency. The host terminal is a mobile application (app) running on a smartphone. Users can send and receive control signals via the mobile device to generate various square wave signals with different characteristics, thereby controlling various movements of the robot. The flexible circuit board has a smaller size and lighter weight. The power supply unit is a lithium battery (220 mAh, 2.93 g). Table S1 lists the detailed characteristic parameters of the wireless circuit board.

The relationship between the forward and backward velocities and excitation frequency of the built-in integration robot is shown in Fig. 4B and C, respectively. The speed variation follows the same pattern as the built-in actuation robot, with an optimal excitation frequency. The maximum forward and backward motion velocity of the robot is 263 and 317.3 mm/s, and the corresponding frequencies are 21.76 and 21.8 kHz, respectively. The speed variation patterns of forward and backward movements are consistent. There is a difference in the optimal excitation frequency and maximum velocity between the tethered and untethered robots, and the main reasons are as follows. (a) We separately fabricate the built-in actuation robot and the built-in integration robot for testing. The tethered and untethered characteristic tests are not conducted on the same robot in different states, but rather 2 different robots. Thus, the fabrication and processing errors cannot be ignored; (b) The effects of the wires on robot motion cannot be ignored due to the light weight; (c) The built-in integration circuit board and battery have an inherent weight, and the control board’s power is lower than that of the external power supply. However, the optimal frequency (difference: less than 1%) and the optimal speed (speed magnitude: ~300 mm/s) under the same voltage for both robots show minimal difference, indicating that the proposed built-in integration method achieves built-in integration and miniaturization without impacting performance. We calculate the CoT (cost of transport) of the built-in actuation robot (18.98) and the built-in integration robot (4.28) (Note S7). We compare them with insects [25–34] in nature and similar centimeter-scale robots [7–9,12,19,21,22,35–41] (Fig. S7). The results indicate that the built-in integration robot have lower movement costs, even lower than some insects.

We conduct an endurance test on the built-in integration robot using a circular track, as shown in Fig. 4D. The robot is equipped with an external guide assistance mechanism consisting of 4 bearings, allowing the robot to move in a continuous loop. The battery capacity is measured at regular intervals. The robot maximum endurance is 32 min, during which the battery voltage drops from 3.92 to 3.17 V (see part II in Movie S5). At this point, the built-in integration robot stops moving and loses control signals, as shown in Fig. 4E.

As the built-in integration robot has limited small enclosed space, heat dissipation is a key issue to ensure performance. We optimize the circuit layout and use thermal silicon pads for heat dissipation, while also incorporating an overheat warning function in the mobile terminal (for details, see Note S6). During the endurance test, the robot’s real-time temperature is simultaneously measured using an infrared thermal imager (UTi380, UNI-T, China). The robot starts at a room temperature of 22.9 °C. The highest temperatures measured at the 10th and 20th minute and at the end are 30.2, 33.7, and 45.5 °C, respectively. The total temperature rise remains within a controllable range of 22.6 °C, as shown in Fig. 4F. At the end of the endurance test, the main heat sources of the robot are concentrated inside the body, while the temperature of most metal components remain around 35 °C. This indicates that the robot experiences minimal thermal impact during practical use. More importantly, the robot is typically used in conjunction with payload platforms, which further help to isolate heat transfer. This is attributed to the development of the wireless motion system and the heat dissipation design. Additionally, the robot does not operate continuously during actual application. Therefore, the actual temperature rise will not exceed the maximum test temperature.

### Application demonstration of the robot

**Fig. 5.**
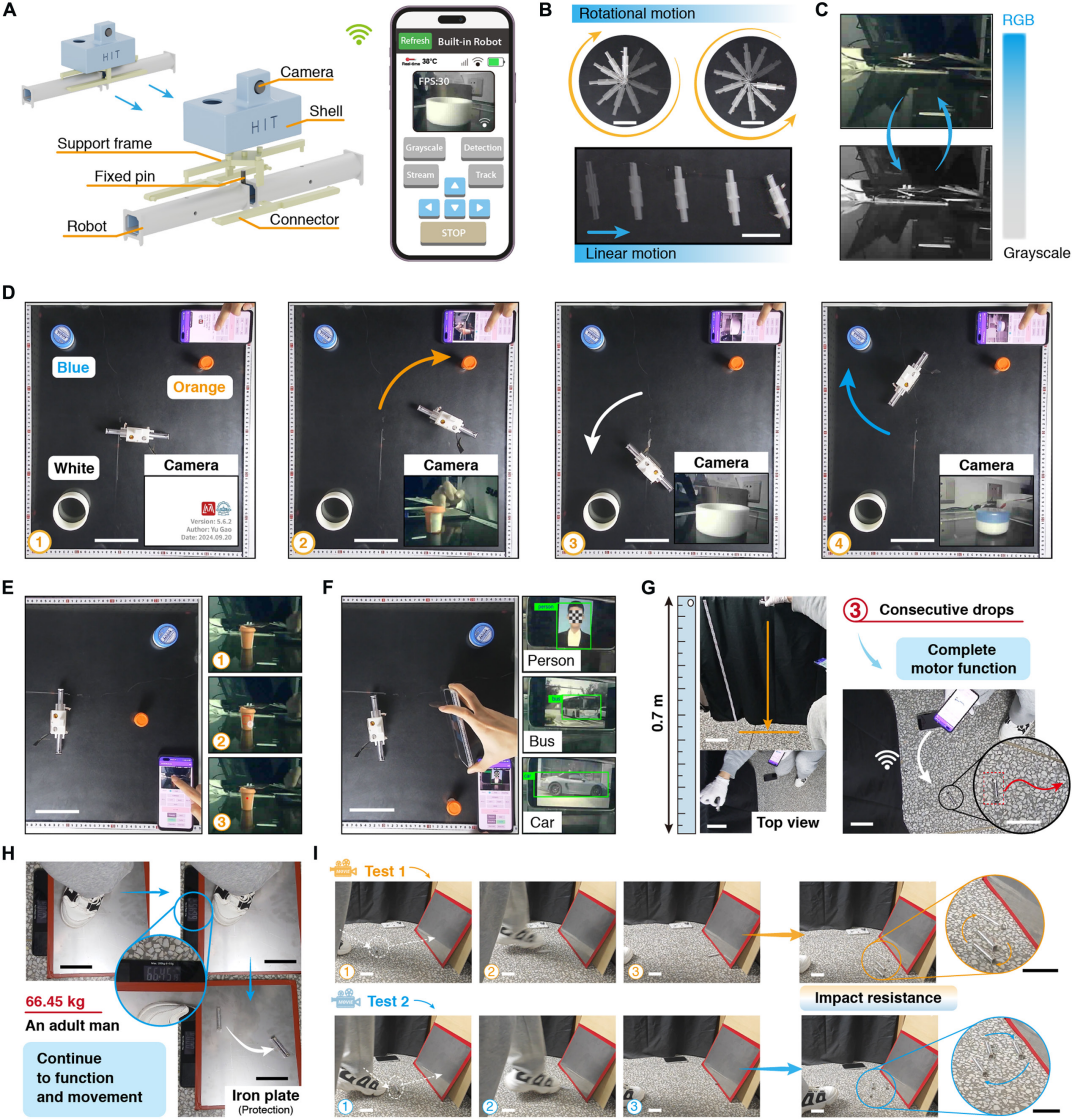
Modular expansion and high robustness application experiments. (A) The structure of the dual-module sensing robot and its corresponding mobile control application. (B) Display of the linear motion and the rotational motion process of the DMSR. Scale bars, 10 cm. (C) Real-time image grayscale conversion (above: RGB, below: grayscale image). (D) Wireless multi-object image capture, and the bottom-right corner shows the captured images from the camera. (1: initial state, 2: the orange object, 3: the white object, 4: the blue object). Scale bars, 10 cm. (E) Real-time color block tracking (1: initial state, 2: select tracking color, 3: real-time display of its center point). Scale bars, 10 cm. (F) Object detection (more than 100 categories including person, bus, and car). Scale bars, 10 cm. (G) In the 3 repeated drop tests, the robot consistently maintains complete wireless mobility capabilities (height: 0.7 m). Scale bars, 10 cm. (H) The robot continues to function after being stepped on by an adult human (66.45 kg), a load about 3,500 times its own body weight. Scale bars, 10 cm. (I) In 2 kick tests, the robot demonstrates good impact resistance (protective plate material: iron plate). Scale bars, 10 cm.

The robot has no external modules and offers enough space for modular expansion. Above the 2 parallel-connected built-in integration robots, a sensor module is installed, forming the DMSR, as shown in Fig. 5A. The sensor module includes a camera, a control module (the control chip: ESP32-S3, Espressif Systems), and a shell. The sensor module and the robot assembly are fixed using a 3D printed support frame and a fixed pin. The DMSR achieves real-time image transmission, image processing, and motion signal transmission and reception through a mobile control application. It is worth noting that the onboard sensor is only responsible for image capture and transmission, while all image processing tasks have been offloaded to the mobile terminal. This approach helps to fully leverage the computational power of the smartphone and reduce the robot’s power consumption. Both the robot and the sensor module communicate and transmit data to the mobile device via 2.4 GHz Wi-Fi. The DMSR is capable of linear and rotational motions through the application, as shown in Fig. 5B (see part I in Movie S6). When DMSR moves in opposite directions on the left and right sides, rotational motion is achieved, with the minimum turning radius. Therefore, each built-in integration robot has only linear motion function, simplifying the control signals to 2 channels. The image processing algorithms are deployed on the mobile terminal, and a series of tests are conducted in image sensing applications by incorporating DMSR. By applying OpenCV algorithms, the images acquired by the robot are converted in real time between RGB and grayscale formats on the mobile terminal, as shown in Fig. 5C (see part II-1 in Movie S6). We place 3 objects (blue, orange, and white) at different positions on the work surface. Using the flexible motion capabilities (controllable linear and turning motions) of DMSR, we search for and capture images of the 3 objects, as shown in Fig. 5D (see part II-2 in Movie S6). Besides, the mobile control terminal of the DMSR also has the ability to track color blocks, as shown in Fig. 5E. First, control the robot to move in front of the color block to be captured. On the mobile control application, mark the color of the block to be captured. The system will calculate the center points of all pixels within the selected color range and mark them with a red dot on the real-time image (see part II-3 in Movie S6). Furthermore, based on TensorFlow Lite, the system is capable of object detection for over 100 categories of objects, as shown in Fig. 5F (see part II-4 in Movie S6). We use photos instead of physical objects to demonstrate the system’s ability to recognize a person, a bus, and a car. These tests highlight the robot’s potential for applications in image detection and modular expansion. Moreover, all computational power is deployed on the mobile device rather than the embedded system, which substantially reduces the robot’s hardware costs and enhances functional expansion.

High robustness is more conducive to the practical application of untethered centimeter-level robots. We simulate a scenario where the robot accidentally falls from the workbench, as shown in Fig. 5G. The both ends of the robot is temporarily sealed and a free fall impact test from the workbench surface is conducted. The height is approximately 0.7 m, and the test is repeated 3 times consecutively. After each test, we verify whether the robot could respond to wireless motion instructions. The results show that after each drop, the robot retain full motion and communication capabilities (see part I in Movie S7).

Besides, the built-in integration robot can continue to function after being stepped on by an adult human (66.45 kg), a load about 3,500 times its own body weight (see part II in Movie S7), as shown in Fig. 5H. We simulate a scenario where the robot is kicked off the ground, as shown in Fig. 5I. The robot is kicked and moves forward, stopping after hitting the iron plate. After 2 kicking impacts, the built-in integration robot still has motion functions, demonstrating its excellent impact resistance (see part III in Movie S7).

We conduct the motion speed characterization experiment on the untethered robot (under 30 *V*_0-p_ conditions) and compare the results with the original untethered motion data (see Fig. S8). It should be noted that during the robustness demonstration experiment, the same robot is subjected to 3 consecutive drops, 2 kicks, and being stepped on by an adult. It can be observed that the maximum forward and backward motion speeds decreased by 28.75% and 16.7%, respectively, which falls within the expected range. The frequencies corresponding to the maximum motion speeds increased from 21.76 to 21.90 kHz (forward) and from 21.80 to 21.84 kHz (backward), corresponding to increases of 0.64% and 0.18%, respectively. The possible reasons are as follows. (a) After the consecutive drops, heavy loading, and impact events, the driving feet of the robot bear most of the force. The tips of these feet inevitably experience wear and deformation, which contributes to the reduction in motion speed and the shift in optimal excitation frequency; (b) Another possible reason is partial damage to the piezoelectric ceramics caused by the strong impacts. Overall, the degradation in the robot’s locomotion performance after enduring important impacts and heavy loads remains within an acceptable range, demonstrating its robustness.

In short, the above experiments prove that the robot has high robustness, which is attributed to the proposed built-in actuation method and built-in integration method. All the functional components are integrated within the metal body, with no external components or connectors. This indicates that the robot retains a certain level of locomotion capability even when encountering unpredictable impacts, falls, and other disturbances during operation. This robustness is lacking in other centimeter-scale untethered external component robots.

## Discussion

In this work, we propose a new built-in actuation method to achieve miniaturization and actuation integration of the centimeter-level robot. The method uses second-order bending vibration as the operating mode, exciting a multi-dimensional elliptical trajectory at the foot, which accumulates microscopic steps to form macroscopic locomotion. The built-in actuation robot achieves fast locomotion with a speed up to 47.38 BL/s (140 *V*_p-p_), a high carrying capability of 28.96 times self-weight, and a high-resolution of 0.33 μm. It can perform cross-scale movement from sub-micron (micro) to meter (macro) level, which demonstrates its potential applications in fields such as micro-manipulation and micro-transportation. Based on the above, the functional components of actuation, control, communication, and power supply are all integrated within the metal body through the built-in integration method, allowing for untethered movement without the need for external components. The built-in integration robot weighs 18.8 g and achieves a maximum untethered speed of 317.3 mm/s. It can operate continuously for 32 min. The built-in integration robot can complete high-speed movements at a lower cost, which is faster than most centimeter-scale terrestrial robots [7–19,21,22,36,41–50], even over the moving speeds of some insects in nature [28,51–54] (see Table S2). In addition, we also list several key parameters (such as load, endurance, startup voltage, and resolution) (see Table S3) of these robots. The proposed robot features high load capacity, long endurance, low startup voltage, and low power consumption. It can be seen that piezoelectric robots possess advantages in precise motion compared to robots with other types of actuators. The proposed robot exhibits a smaller step size than similar piezoelectric robots. The robot can perform cross-scale movement from sub-micron (micro) to meter (macro) level.

Moreover, the robot demonstrates high robustness and practical application potential. Except for the glass work surface, effective motions are achieved on 6 other contact surfaces. After being stepped on by an adult human’s full body weight (66.45 kg, over 3,500 times heavier than the robot), as well as enduring 3 consecutive drops and 2 kicks, the robot still operates normally and continues to move afterward. Through experiments such as remote multi-object image capture, real-time image grayscale conversion, real-time color block tracking, and object detection, the built-in integration robot demonstrates its potential for image sensing applications and modular expansion.

Although the proposed robot demonstrates excellent performance in certain aspects, it still has some limitations: (a) The robot and the sensor module are fixedly connected, which limits the ability to switch functions. A simpler connection method would facilitate easier module expansion and application; (b) The flexible circuit board outputs a low voltage (0 to 30 *V*_0-p_). Increasing its output power and maximum voltage can enhance the performance; (c) Since the robot is primarily designed for applications such as micro-manipulation platforms and point-to-point positioning systems, its motion typically occurs on flat surfaces, making it difficult to adapt to uneven or bumpy working environments. Enhancing the robot’s environmental adaptability would help expand its range of applications.

## Conclusion

We propose a new built-in actuation method and a centimeter-scale compact robot prototype is designed. It achieves fast locomotion, a high carrying capability, and high-resolution motion. The robot can perform cross-scale movement from sub-micron to meter level. The untethered robot realizes the integration of actuation, control, communication, and power supply through the built-in integration method, ensuring all functional units are protected within the metal substrate of the piezo unit. We demonstrate through a series of tests that the robot has certain robustness performance. The robot with a sensor module achieves real-time image grayscale conversion, multi-object image capture, color block tracking, and object detection, exhibiting great potential for image sensing applications and modular expansion. The built-in actuation method and the built-in integration method address the 2 main issues faced by the piezoelectric robot: (a) relatively poor robustness; and (b) low integration of the robot. The proposed methods provide a design reference for miniaturization and strong robustness in centimeter-scale robot.

Some possible future directions and challenges include the following: (a) Exploring more efficient built-in actuation methods and piezoelectric ceramic arrangements to improve the motion performance of the robot; (b) Try to integrate small functional modules, such as micro grippers, to achieve cross-scale applications with multiple positioning points, long distances, and micro-gripping capabilities; (c) Increase the robot’s position feedback capability for more precise motion control.

## Data Availability

All data supporting the findings of this study are available in the paper and the Supplementary Materials.

## Supplementary Materials

Supplementary 1 Notes S1 to S7 Figs. S1 to S10 Tables S1 to S3 Movies S1 to S7
