# Supplementary material for: A Centimeter-Scale Quadruped Piezoelectric Robot with High Integration and Strong Robustness
Source: Cyborg Bionic Syst. 2025 Jul 22;6:0340. doi: 10.34133/cbsystems.0340 (PMC12282480; doi:10.34133/cbsystems.0340)
Supplement: Supplementary 1 — Notes S1 to S7 Figs. S1 to S10 Tables S1 to S3 Movies S1 to S7 [file cbsystems.0340.f1.zip › The supplementary materials (Clean Version).docx]

Supplementary Materials for

**A Centimeter-scale Quadruped Piezoelectric Robot with High Integration and Strong Robustness**

Yu Gao1†, Jing Li1†, Jie Deng1†, Shijing Zhang1, Yingxiang Liu1*

Corresponding author: Yingxiang Liu, [liuyingxiang868@hit.edu.cn](mailto:liuyingxiang868@hit.edu.cn)

**Affiliations:**

1State Key Laboratory of Robotics and System, Harbin Institute of Technology, Harbin, 150001, China.

†These authors contributed equally to this work

*Corresponding author. Email: [liuyingxiang868@hit.edu.cn](mailto:liuyingxiang868@hit.edu.cn) (Y.L.)

**The PDF file includes:**

**Supplementary Note 1 to Note 7.**

Note 1: The comparison between built-in and external piezoelectric ceramics.

Note 2: Material parameters of the robot.

Note 3: Experimental set-ups for the vibration characteristic of the robot.

Note 4: Test system for robot vibration displacement.

Note 5: Test system for robot motion resolution.

Note 6: Design of motion control system.

Note 7: CoT evaluation of the robot.

**Supplementary Fig. 1 to Fig. 10.**

Fig. S1: The comparison between built-in and external piezoelectric ceramic plates.

Fig. S2: The key structural parameters and weight of each unit.

Fig. S3: Experimental test system for the vibration mode.

Fig. S4: Vibration displacement test system based on laser displacement sensor.

Fig. S5: Test system for the robot motion resolution.

Fig. S6: The schematic diagram of control circuit system and circuit board structure.

Fig. S7: CoT comparison of the proposed robot with other similar robots and insects.

Fig. S8: Untethered motion speed comparison (30 V0-p) after the robot endured three consecutive drops, two kicks, and being stepped on by an adult (over 3500 times its own weight).

Fig. S9: The fabrication processes.

Fig. S10: Robot locomotion test platform.

**Supplementary Table 1 to Table 2.**

Table S1: Characteristic parameters of the wireless circuit board.

Table S2: Comparison of relative speed and mass with other similar robots and insects.

Table S3: Comparison of key parameters with other similar robots.

**Other supplementary information for this manuscript includes the following:**

**Supplementary Movies 1 to 7.**

Movie S1: The configuration and structure of the robot.

Movie S2: The principle of the built-in actuation method.

Movie S3: Robot vibration mode test.

Movie S4: Locomotion characteristics of the built-in actuation robot.

Movie S5: Locomotion characteristics of the built-in integration robot.

Movie S6: Robot extensibility and applications.

Movie S7: Robot robustness test.

**Supplementary Note 1:**

**The comparison between built-in and external piezoelectric ceramics.**

The polarization direction of the piezoelectric ceramic is along the thickness direction, and the excitation signal is applied from the silver layers on both sides. The piezoelectric ceramic extends or contracts based on the excitation signal by the inverse piezoelectric effect. In the actuation mode, the piezoelectric ceramic is generally pasted on the surface of the metal body.

**Supplementary Fig. 1** shows the actuation principle of different installation methods of piezoelectric ceramics. Both external and built-in installation methods can achieve deformation of the elastic body. But the built-in installation method has many advantages. In the built-in installation method, the excitation signals on both sides of the two opposing ceramics inside are consistent, which helps optimize the circuit layout and also provide the possibility of the built-in control system. However, the wires for the external installation method need to span across the metal body and two ceramic pieces. Furthermore, the robot with built-in-ceramics can protect the actuation sources and crucial components, reducing wear and providing features such as dust and water resistance. The built-in structure provides a more compact structure and the hollow structure significantly helps reduce resonant frequency and increase resonant amplitude.

**Supplementary Note 2**

**Material parameters of the robot**

The robot prototype is completed by CNC and laser cutting, and the material is aluminum alloy (2A12, Density of 2.77×103 kg/m3, Young’s modulus of 7.1 × 1010 N/m2, and Poisson’s ratio of 0.33). The piezoelectric ceramic plates are made of PZT-4 (Lead Zirconate Titanate, Density of 7.6×103 kg/m3). Other physical parameters of PZT-4 obtained from the supplier are as follows:

where , and are the stiffness matrix, the piezoelectric matrix, and the dielectric matrix, respectively.

**Supplementary Note 3**

**Experimental set-ups for the vibration characteristic of the robot**

The laser vibrometer test system is shown in **Supplementary Fig. 3**. The vibration mode of the robot is measured by using a scanning laser Doppler vibrometer (PSV-400-M2, Polytec, Germany). The instrument mainly includes a scanning laser head, a vibrometer controller, a power amplifier (ATA-4051, Aigtek Inc., China) and data management system. The vibrometer controller can generate sinusoidal signals from 0 to 1000 kHz, the power amplifier is used to amplify the voltage. The scanning laser head can generate a laser, which is vertical to the measured plane, and the tested data is stored in the data management system. The host computer sends a start signal to the vibrometer controller, which generates a series of sinusoidal signals and inputs them to the robot through the power amplifier. The laser generator sweeps the frequency of the robot that is vibrating continuously. Finally, the vibration shape of the robot is obtained on the host computer.

**Supplementary Note 4**

**Test system for robot vibration** **displacement**

The robot vibration displacement testing system is designed to capture micron-level vibration displacements of the robot driving foot, as shown in **Supplementary Fig. 4**. Two stainless steel rods are inserted into the middle hole on the robot base, and two precision flat pliers are used to fix the robot in the OX and OY directions respectively. Since the end of the robot foot is sharp and cannot reflect the laser spot, we equivalently capture the displacement values of the robot foot-end by measuring two adjacent points on the top and side of the robot. The robot is powered by an ultrasonic power supply (QD-8D, China), and two laser displacement sensors (LK-H020, Keyence, Japan) are used to test the response displacements on a vibration isolation platform.

**Supplementary Note 5**

**Test system for robot motion resolution**

We have built a robot resolution testing system, as shown in **Supplementary Fig.5a**. The built-in actuation robot is powered by an external ultrasonic power supply and response displacement with high resolution is measured by using capacitive displacement sensors (D-E20.050, Physik Instrumente, Germany).

The high-resolution mode of the robot is achieved through high-frequency pulse excitation. A high-frequency pulse signal is composed of a series of high-frequency sine signals with certain intervals. During the testing, the voltage of the sinusoidal signal is held constant at 15 Vp-p, with a frequency of 21.98 kHz. The duty cycle of the pulse is 1%, which means that there is a high-frequency sine signal during the 1% time period of the cycle. By adjusting the period of the pulse signal (10-100 Hz) to change the step distance of the robot.

The signal acquisition modules are used to convert digital signals and analog signals. Finally, the data is transmitted back to the host computer. The robot is equipped with an extension board with copper foil, and the experiment uses two parallel sensor probes for testing to eliminate rotation errors. The final motion resolution is the average of the two sensor test results.

**Supplementary Note 6**

**Design of motion control system**

The robot possesses a built-in flexible circuit board with a weight of only 0.83 g to provide excitation signals to achieve untethered locomotion. The circuit board can achieve four independent square wave signal outputs, with a maximum output voltage of 30 V0-p. The remaining detailed parameters are shown in **Supplementary Table 1**. The schematic diagram and layout diagram of the control board are shown in **Supplementary Fig. 6**. The control chip is ESP32-PICO-D4 (Espressif, China), which provides communication and control signals.

Positive high voltages are generated by two single-chip high-frequency switching regulators (TPS61170) and transformers (ATB322515-0110), which are provided to the AC boost circuit. The negative voltage generation circuit uses the charge pump principle and the low-noise regulated inverter chip LM27761 to generate a -3.3V voltage, which serves as the negative power supply input for the amplifier. The high-voltage operational amplifier (LTC6091) in the circuit generates the corresponding high-frequency and high-voltage output by comparing the DC bias voltage provides in the reference voltage circuit (provided by LMV321, 1.65V) with the pin voltage of the controller. The module is powered by a lithium battery (3.7 V, 2.93 g, 220 mAh, YuHuiDa, Inc.). To improve heat dissipation, high-temperature components such as transformers and the control chip are placed at opposite ends of the circuit board. Thermal silicone pads are applied to critical chips to further enhance thermal management.

In addition, the control board is equipped with active and passive overheat protection functions. Passive protection: The operational amplifier chip is equipped with a temperature monitoring pin, which can automatically disable the amplifier output when the operational amplifier chip reaches 140℃. Active protection: By real-time reading of the temperature sensor embedded in the control chip (ESP32-PICO-D4), the internal temperature of the robot body is monitored. When it reaches 70℃, an alarm is sent to the user through a mobile terminal. When it reaches 80℃, the boost output is cut off and the user is notified.

**Supplementary Note 7**

**CoT evaluation of the robot**

The cost of transport (CoT) is commonly used in biology to assess the locomotion cost of insects and is also employed for evaluating the motion energy consumption of robots. It is determined by the following formula:

(1)

where is the instantaneous power consumption, is the weight of the robot, is the gravitational acceleration, and is the robot speed. We utilize a digital power meter (WT210, Japan) to measure the power consumption of the built-in integration robot under untethered locomotion conditions (30 V0-p), achieving a motion speed of 317.3 mm/s. The power consumption of the module is 0.25 W. Thus, the CoT reaches 4.28. The power consumption of the built-in actuation robot under tethered locomotion conditions (140 Vp-p) is 1.658 W. The movement speed of the robot reaches 616 mm/s. Thus, the CoT reaches 18.98. It can be seen that the power required for the untethered actuation is low, which is due to the low starting voltage of the robot. However, the low driving power of the wireless control board will still have a negative effect on the motion effect of the robot.

We conduct a simple comparison with the CoT of some centimeter-scale robots and some insects, as shown in **Supplementary Fig. 8**. Note: the partly CoT values are not explicitly published in the papers. They are calculated to the best of our ability using the available data. The proposed robot exhibits lower motion costs, especially under the untethered motion condition.


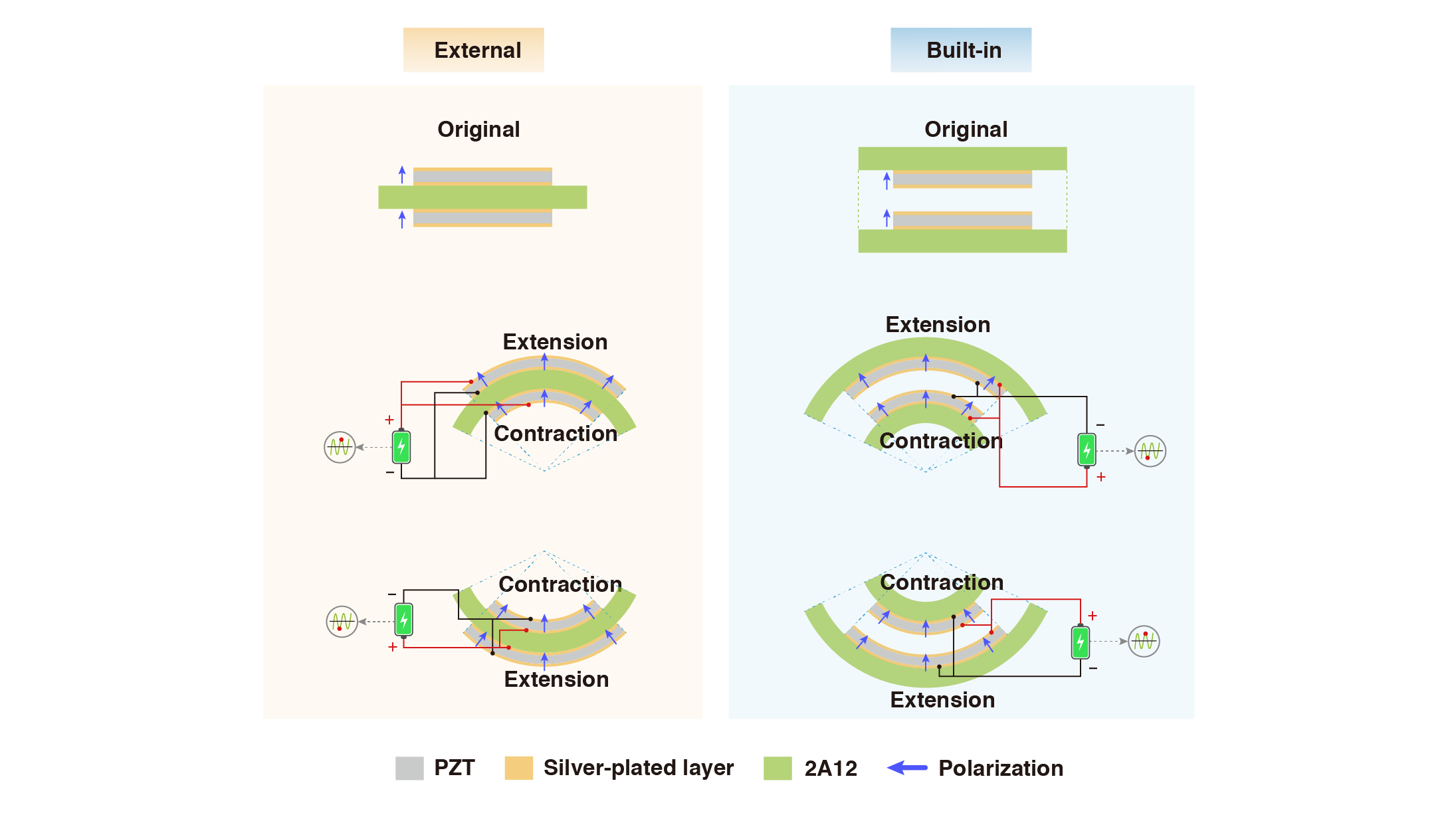


**Supplementary Fig. 1.**

**The comparison between built-in and external piezoelectric ceramic plates.**


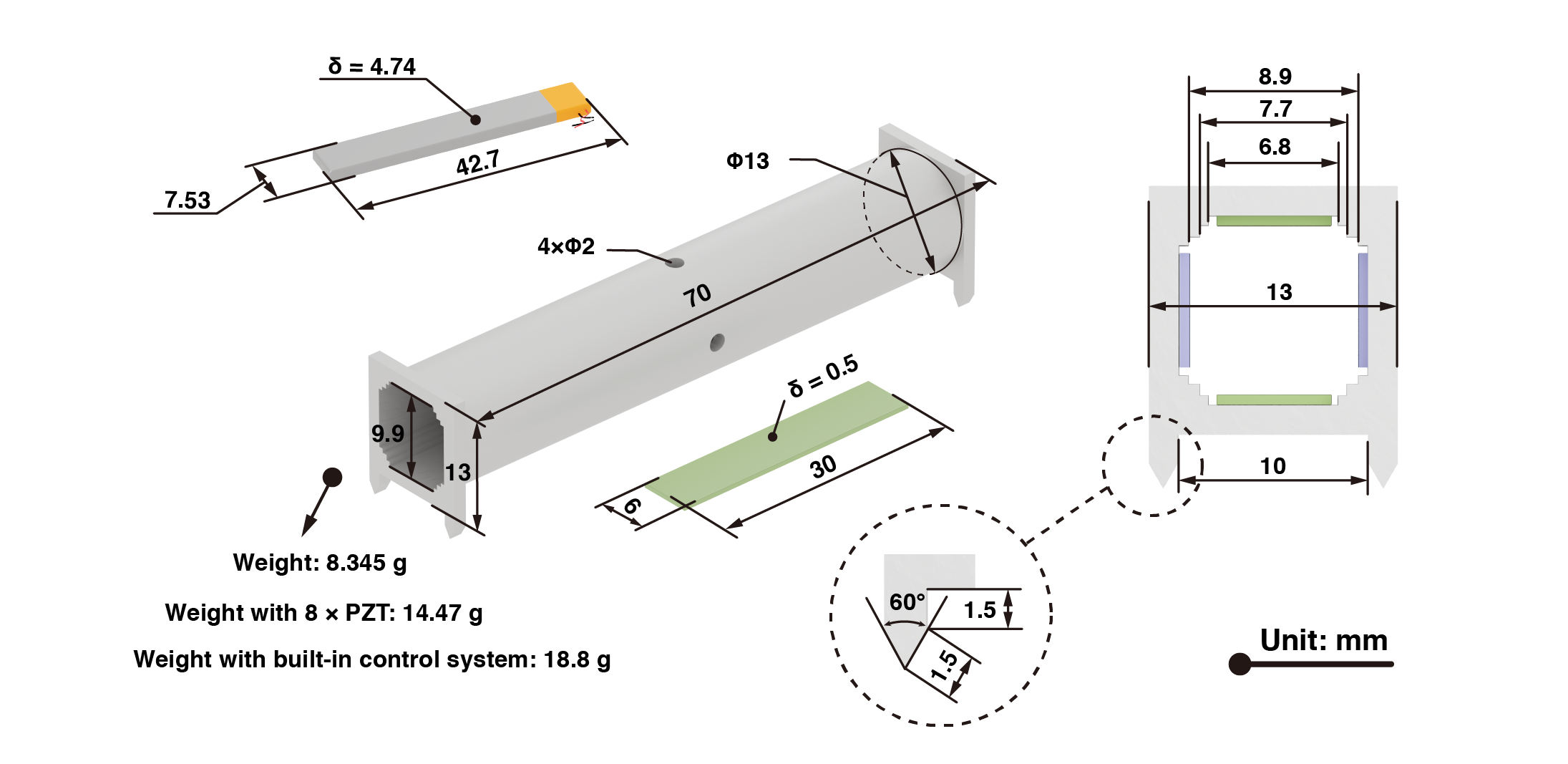


**Supplementary Fig. 2.**

**The key structural parameters and weight of each unit.**


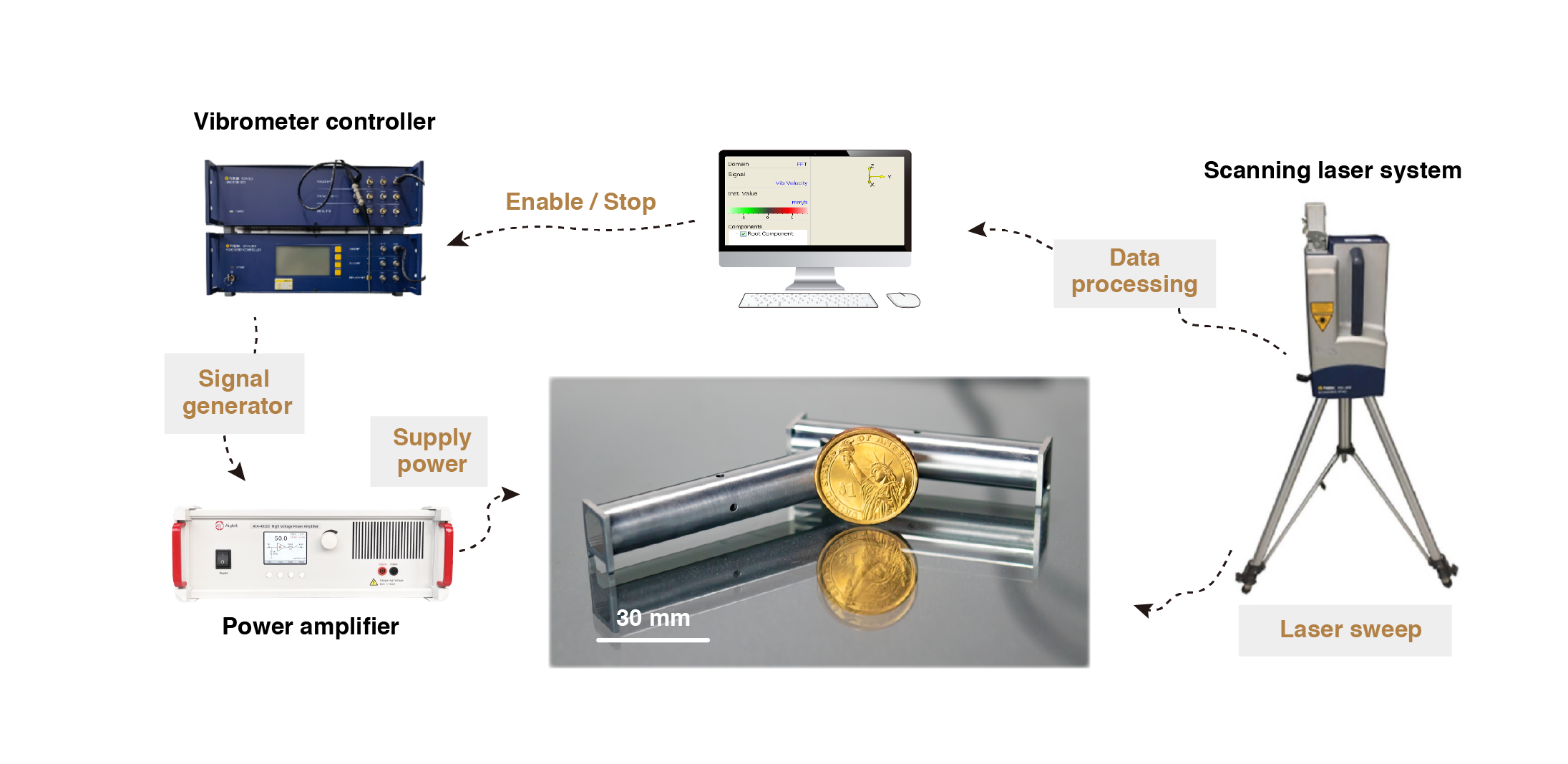


**Supplementary Fig. 3.**

**Experimental test system for the vibration mode.**


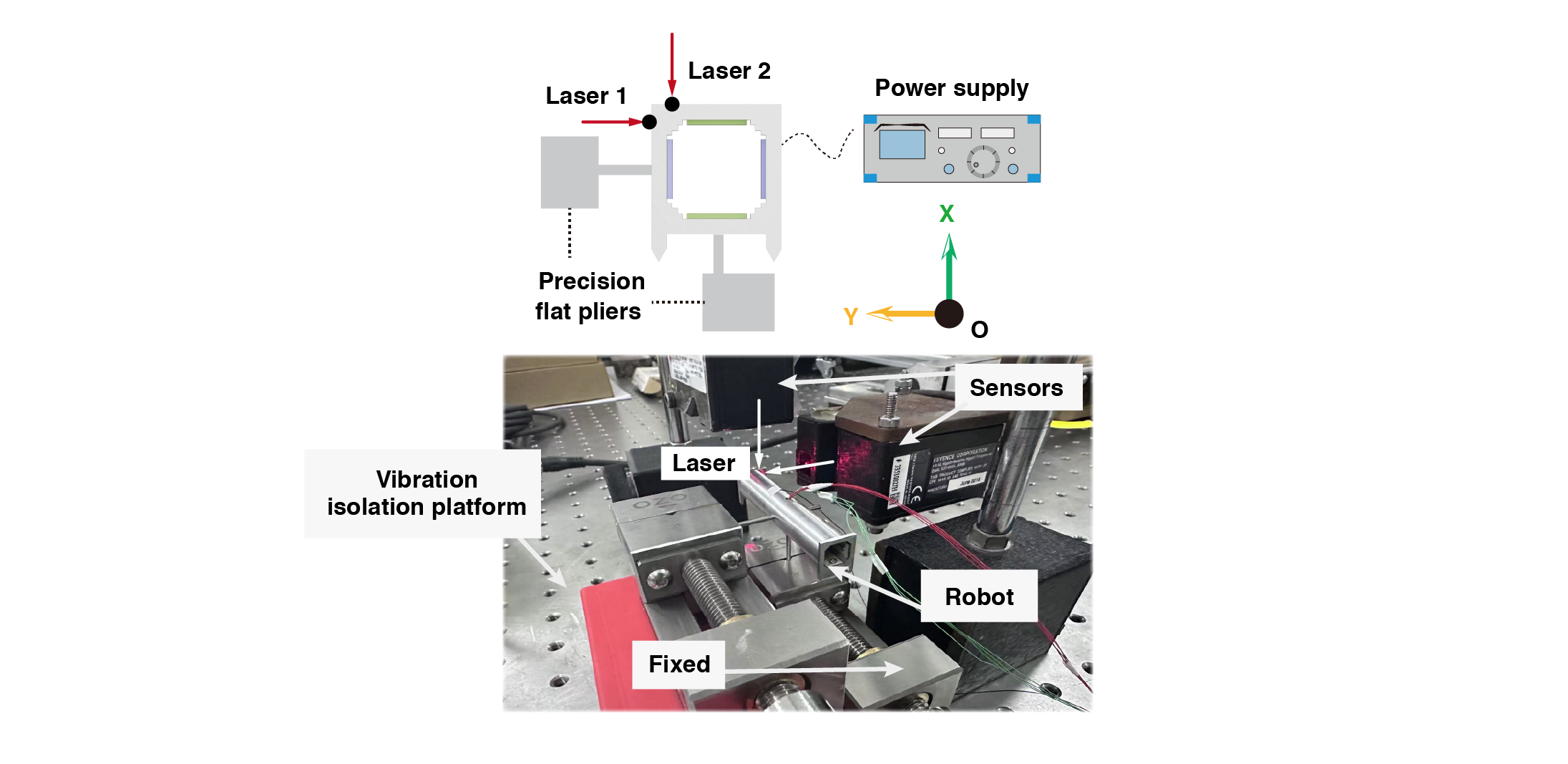


**Supplementary Fig. 4.**

**Vibration displacement test system based on laser displacement sensor.**


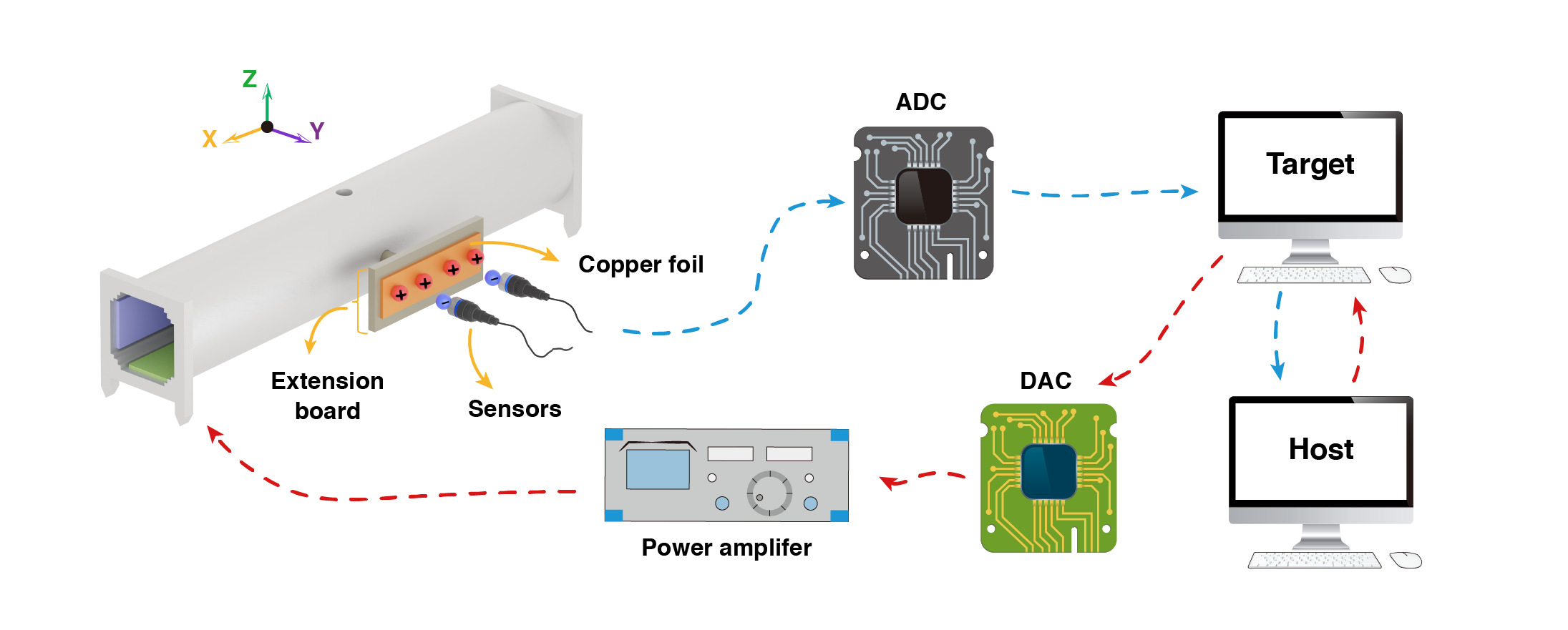


**Supplementary Fig. 5.**

**Test system for the robot motion resolution.**


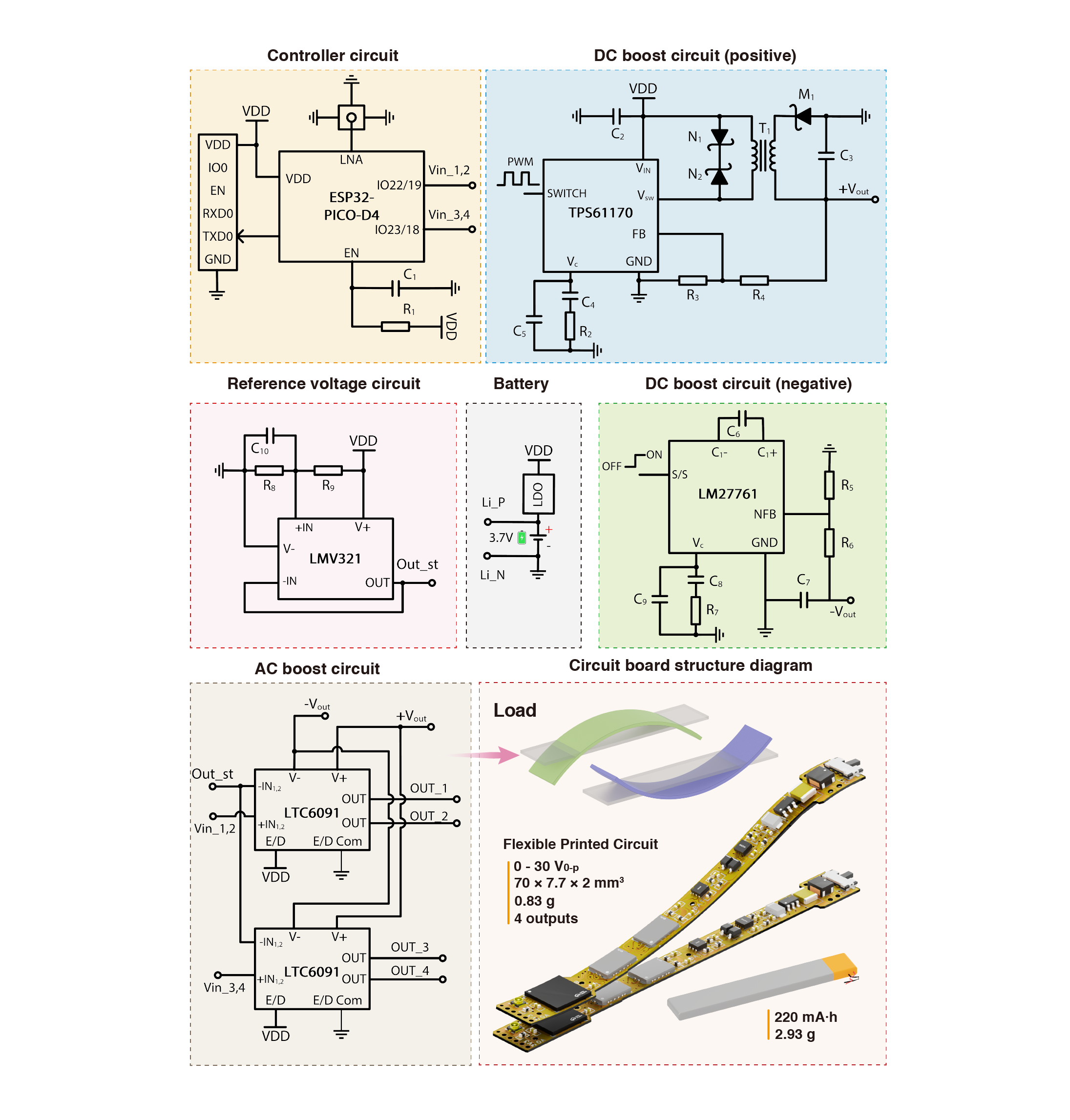


**Supplementary Fig. 6.**

**The schematic diagram of control circuit system and circuit board structure.**


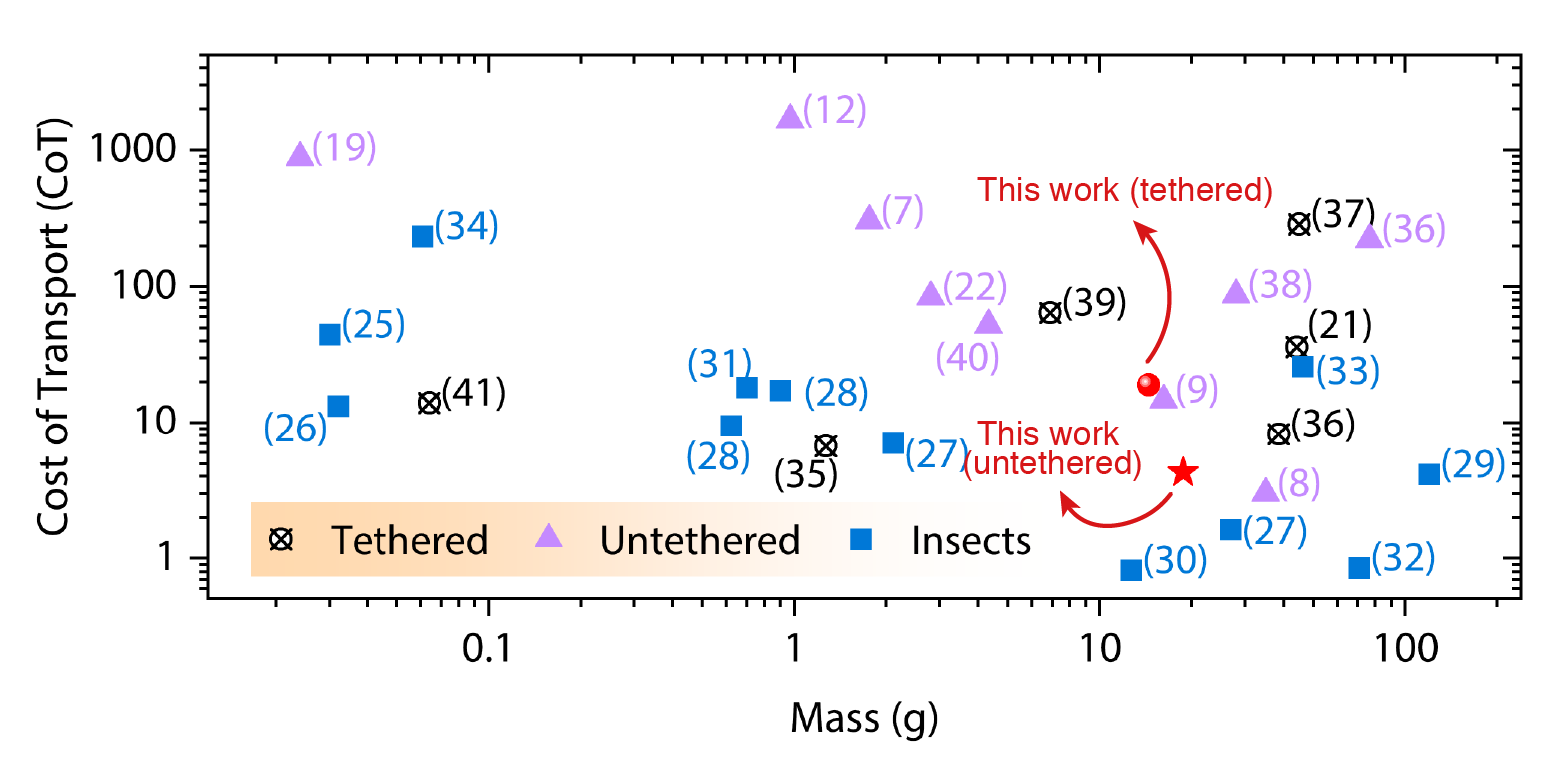


**Supplementary Fig. 7.**

**CoT comparison of the proposed robot with other similar robots and insects.**

Note: Part of the CoT data is not directly provided and is calculated from the known data in the paper. The numbers near the symbols identify the source of the reference.


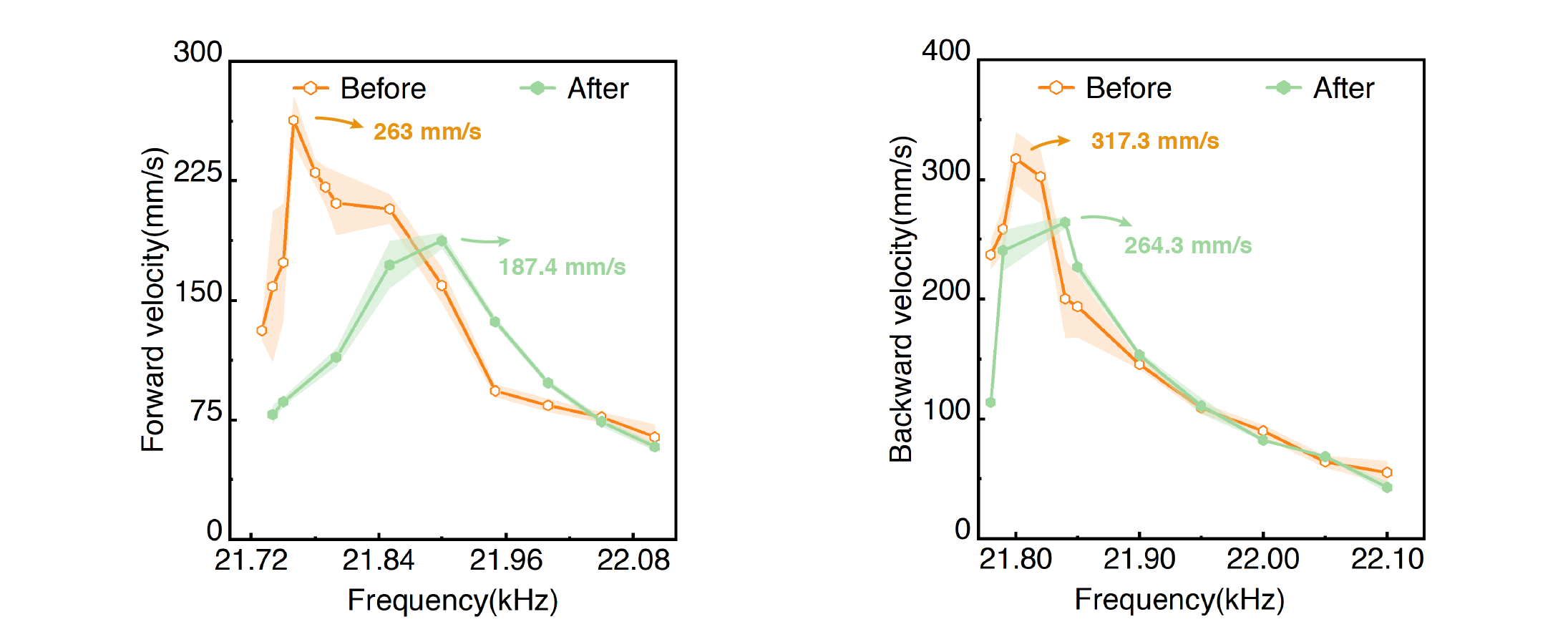


**Supplementary Fig. 8.**

**Untethered speed comparison (30 V0-p) after the robot endured three consecutive drops, two kicks, and being stepped on by an adult (over 3500 times its own weight).**


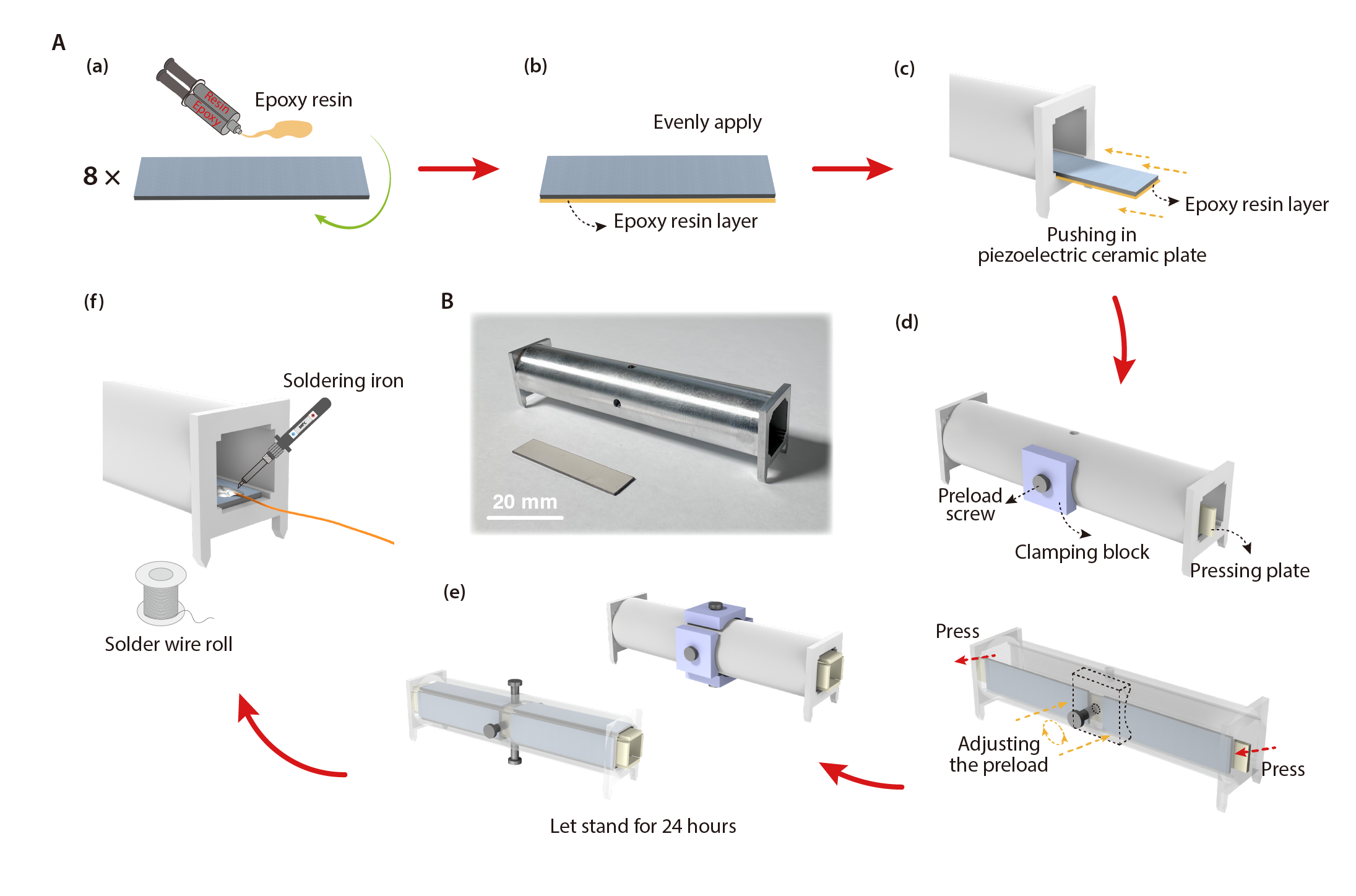


**Supplementary Fig. 9.**

**The fabrication processes. (A)** The fabrication processes. **(B)** The photo of the metal body and a piece of piezoelectric ceramic.


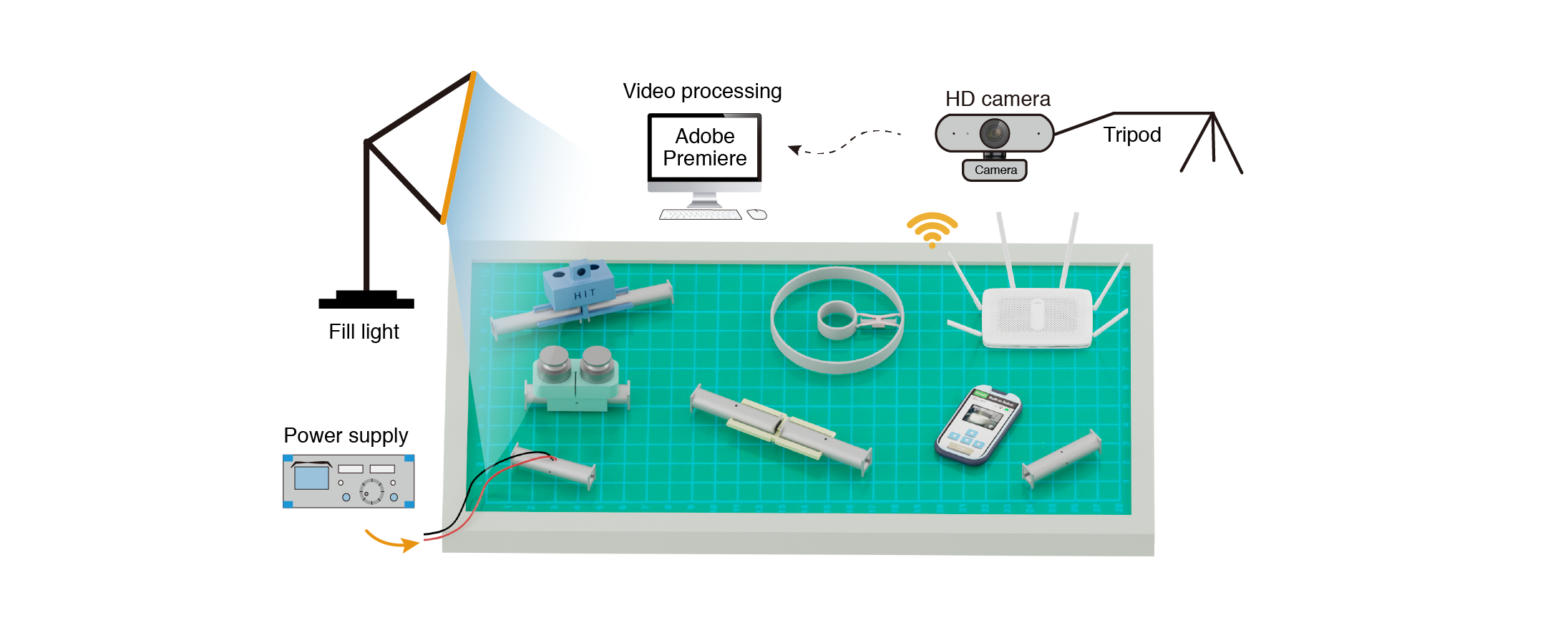


**Supplementary Fig. 10.**

**Robot locomotion test platform.**

**Supplementary Table 1**

**Characteristic parameters of the wireless circuit board**

| Characteristics | Parameters |
| --- | --- |
| Size (mm3) | 70 × 7.7 × 2 |
| Weight (g) | 0.83 |
| Wave type | Square wave |
| Output channels | 4 (independent) |
| Bandwidth (kHz) | 0 - 50 |
| Voltage (V0-p) | 0 - 30 |
| Ripple (mVp-p) | 200 - 400 |
| Control chip | ESP32-PICO-D4 |
| Communication mode | 2.4 GHz Wi-Fi |
| Power (W) | 0.25 (30 V0-p) |

**Supplementary Table 2**

**Comparison of relative speed and mass with other similar robots and insects**

| **Species** | **Size (mm3)** | **Mass (g)** | **Relative speed**  **(mm/s) / (BL/s)** | **Reference** |
| --- | --- | --- | --- | --- |
| **Insect** |  |  |  |  |
| Stagmomantis theophila (1st Instar) | 12.6 ± 0.2 | 0.0068 | 650 / 51.6 | *(54)* |
| Stagmomantis theophila (4th Instar) | 26.3 ± 0.7 | 0.0505 | 920 / 35.0 | *(54)* |
| Stagmomantis theophila (Adult male) | 59.2 ± 1.4 | 0.3133 | 1060 / 17.9 | *(54)* |
| Formica fusca | / | 0.005 | 26.9 / - | *(52)* |
| Onymacris plana | ~ 20 | 0.73 | 1000 / 50 | *(51)* |
| Periplaneta americana | / | 0.90 | - / 40 | *(28)* |
| Schizocosa ocreata | / | 0.05 | 500 / - | *(53)* |
| **Robots (*tethered*)** |  |  |  |  |
| **Piezoelectric** | **70×13×15.8** | **14.47** | **616 / 47.38** | **This work** |
| Piezoelectric | 30×15× / | 0.024 | - / 20 | *(41)* |
| Piezoelectric | 186×186×86.5 | 1282 | 712 / 3.83 | *(45)* |
| Piezoelectric | 30×15× / | 0.065 | - / 7.81 | *(19)* |
| Piezoelectric | 58×44×12 | 44.25 | 516.3 / 8.9 | *(21)* |
| Dielectric elastomer | / | < 0.2 | - / 1.03 | *(11)* |
| Dielectric elastomer | 100×70×100 | 9.6 | 78 / 0.78 | *(42)* |
| Dielectric elastomer | 91 (Length) | 4.8 | 124 / 1.36 | *(10)* |
| Pneumatic | 70 (Length) | 45 | 187.6 / 2.68 | *(43)* |
| Pneumatic | 60 (Diameter) | 70.2 | 20.1 / 0.201 | *(44)* |
| Shape memory alloy | 42×6×4 | 0.61 | 0.15 / - | *(14)* |
| Magnetic | ~ 3 (Length) | 0.005 | 213 / 57.6 | *(15)* |
| Electromagnetic | 9×9×0.8 | 0.096 | 630 / 70 | *(16)* |
| Light-driven | 42×8× / | - | 0.082 / 0.002 | *(17)* |
| Heat-driven | 70×14× / | 0.29 | 0.72 / 0.012 | *(18)* |
| **Robots (*untethered*)** | | | | |
| **Piezoelectric** | **Same size** | **18.8** | **317.3 / 24.4** | **This work** |
| Piezoelectric | 45 (Length) | 2.8 | 172 / 3.8 | *(22)* |
| Piezoelectric | 31×44×20 | 35.2 | 146 / 4.1 | *(49)* |
| Piezoelectric | 24×32× / | 0.38 | 60 / 2.5 | *(46)* |
| DC motor | 100 (Length) | 16 | 1500 / 15 | *(9)* |
| DC motor | 75×95×21 | 35 | 815 / 10.9 | *(8)* |
| DC motor | 90 (Length) | 146 | 900 / 10 | *(48)* |
| DC motor | 30 (Diameter) | 36 | 10 / 0.3 | *(47)* |
| Shape memory alloy | 15.2 (Length) | 0.088 | 0.76 / 0.05 | *(50)* |
| Shape memory alloy | 30 (Length) | 2.4 | 30 / 1 | *(13)* |
| Dielectric elastomer | 40 (Length) | 0.97 | 12 / 0.3 | *(12)* |
| Electromagnetic | 20 (Length) | 1.76 | 350 / 17.5 | *(7)* |
| Electrostatic | 5 (Length) | 0.0464 | 5.9 / 1.18 | *(36)* |

Note: Some of the data in the table are estimated.

**Supplementary Table 3**

**Comparison of key parameters with other similar robots**

| **Load (times**  **self-weight)** | **Endurance (min)** | **Startup Voltage** | **Resolution**  **(μm)** | | **Reference** | |
| --- | --- | --- | --- | --- | --- | --- |
| **28.96** | **32** | **10 V0-p** | | **0.33** | | **This work** |
| 6 | / | 8 Vp-p | | / | | *(41)* |
| 2.34 | / | 50 Vp-p | | 0.49 | | *(45)* |
| 6.9 | / | / | | / | | *(19)* |
| 4.7 | / | 20 Vp-p | | 0.44 | | *(21)* |
| / | / | Kilovolt level | | / | | *(11)* |
| / | / | ~ 3 kV | | / | | *(42)* |
| / | / | Kilovolt level | | / | | *(10)* |
| 3 | / | / | | / | | *(14)* |
| / | / | ~ 6 Vp-p | | / | | *(16)* |
| / | / | ~ 150 V0-p | | / | | *(22)* |
| 8.7 | 120 | 30 Vp-p | | / | | *(49)* |
| 4.7 | 21.5 | ~ 200 Vp-p | | / | | *(46)* |
| / | 40 | ~ 3.3 V | | / | | *(9)* |
| / | 25 | ~ 3.3 V | | / | | *(8)* |
| / | / | ~ 3.3 V | | / | | *(48)* |
| / | ~ 180 | ~ 3.3 V | | / | | *(47)* |
| 2.61 | / | / | | / | | *(50)* |
| / | / | ~ 13.6 V | | / | | *(13)* |
| 5 | / | ~ 450 V | | / | | *(12)* |
| 5 | 3 | ~ 3.3 V | | / | | *(7)* |
| 4 | / | ~ 1kV | | / | | *(36)* |

Note: Some of the data in the table are estimated. For example, the start-up voltage is not provided in the original text and is calculated based on the minimum voltage reported in the test data.
